# Estimating Demographic Parameters for Bearded Seals, *Erignathus barbatus*, in Alaska Using Close‐Kin Mark‐Recapture Methods

**DOI:** 10.1111/eva.70035

**Published:** 2024-11-08

**Authors:** Brian D. Taras, Paul B. Conn, Mark V. Bravington, Andrzej Kilian, Aimée R. Lang, Anna Bryan, Raphaela Stimmelmayr, Lori Quakenbush

**Affiliations:** ^1^ Alaska Department of Fish and Game Statewide Marine Mammals Juneau USA; ^2^ Marine Mammal Lab NOAA Alaska Fisheries Science Center Seattle USA; ^3^ Estimark Research Hobart Tasmania Australia; ^4^ Diversity Arrays Technology Pty Ltd University of Canberra Bruce Australian Capital Territory Australia; ^5^ Southwest Fisheries Science Center NOAA‐Fisheries La Jolla California USA; ^6^ Alaska Department of Fish and Game Arctic Marine Mammal Program Fairbanks USA; ^7^ Department of Wildlife Management North Slope Borough Utqiaġvik Alaska USA; ^8^ Institute of Arctic Biology University of Fairbanks Fairbanks Alaska USA

**Keywords:** abundance estimation, kinship, life history, phocid, subsistence harvest, survival

## Abstract

Reliable estimates of population abundance and demographics are essential for managing harvested species. Ice‐associated phocids, “ice seals,” are a vital resource for subsistence‐dependent coastal Native communities in western and northern Alaska, USA. In 2012, the Beringia distinct population segment of the bearded seal, *Erignathus barbatus nauticus*, was listed as “threatened” under the US Endangered Species Act requiring greater scrutiny for management assessments. We sought to estimate requisite population parameters from harvested seals by using close‐kin mark‐recapture (CKMR) methods, the first such application for marine mammals. Samples from 1758 bearded seals harvested by Bering, Chukchi, and Beaufort Sea communities during 1998–2020 were genotyped, genetically sexed, and aged by tooth annuli. After rigorous quality control, kin relationships were established for 1484 seals including two parent–offspring pairs (POPs) and 25 potential second‐order kin pairs. Most of the second‐order kin were half‐sibling pairs (HSPs), but four were potential grandparent‐grandchild pairs (GGPs). There were no full sibling pairs, suggesting a lack of mate fidelity. Mitochondrial DNA analysis identified 17 potential HSPs as paternally related, providing substantial evidence of persistent heterogeneity in reproductive success among adult males. The statistical CKMR model incorporates probabilities associated with POPs, HSPs, and GGPs and assumes known ages and a stable population. Our top model accommodates heterogeneity in adult male breeding success and yields an abundance estimate of ~409,000 with a coefficient of variation (CV) = 0.35, which is substantially greater than the “non‐heterogeneity” model estimate of ~232,000 (CV = 0.21), an important difference for managing a harvested species. Using CKMR methods with harvested species provides estimates of abundance with the added opportunity to acquire information about adult survival, fecundity, and breeding success that could be applied to other species of concern, marine and terrestrial.

## 1 Introduction

**FIGURE 1:**
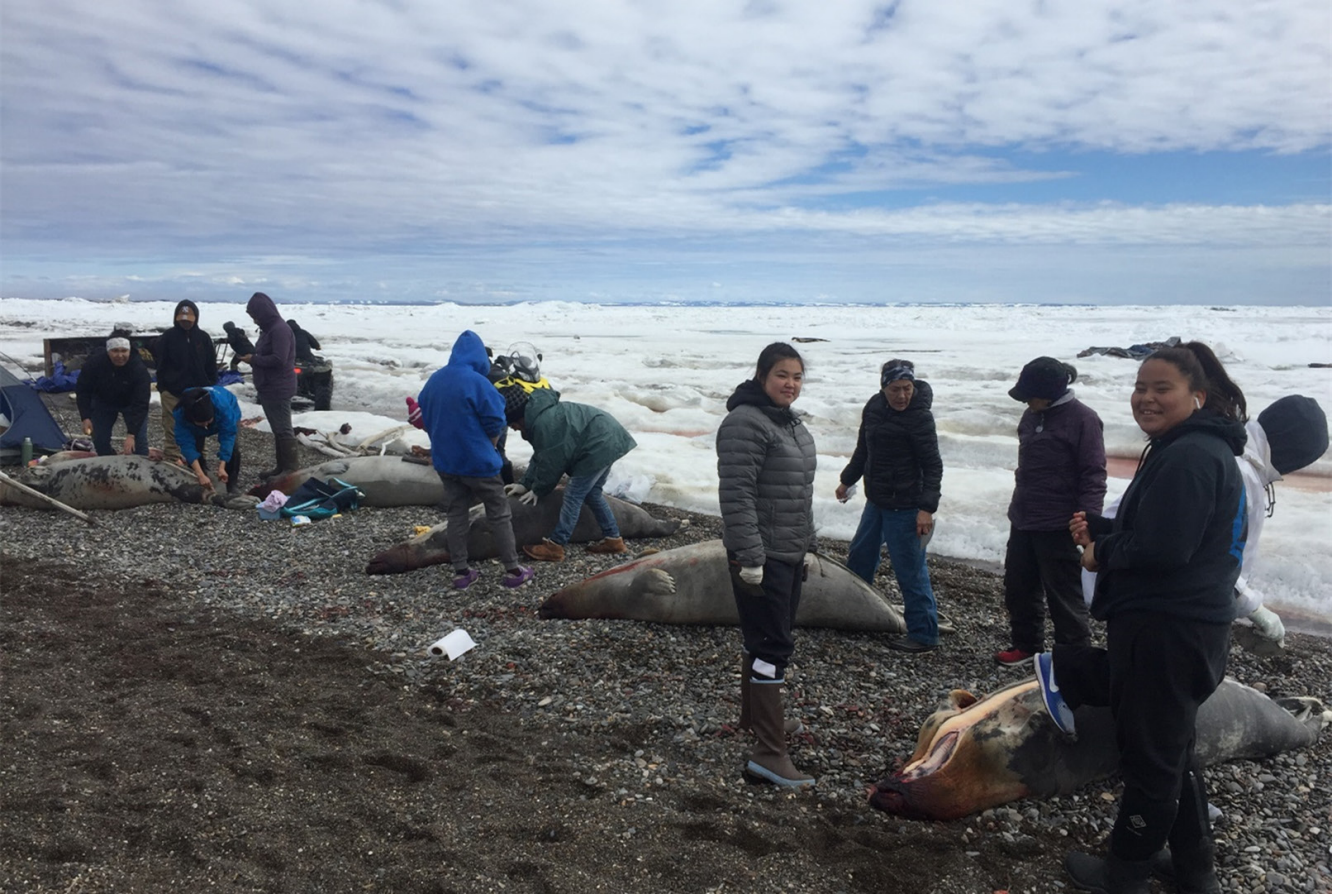
Spring bearded seal harvest at Point Hope, Alaska. Seals are being prepared for use of the skin, meat, and oil. Photo credit Alaska Department of Fish and Game.

Reliable estimates of population abundance and demographics are essential for managing harvested species such as sea ice‐associated phocids called “ice seals.” Ice seals are a vital resource for subsistence‐dependent coastal Native communities in western and northern Alaska, USA, as they are widely used for food, clothes, boat skins, and handicrafts (Figure 1; Fall 2014). All marine mammals in the United States are protected under the Marine Mammal Protection Act. In 2012, the Beringia distinct population segment (DPS) of the bearded seal, *Erignathus barbatus nauticus*, was listed as “threatened” under the US Endangered Species Act due to its reliance on sea ice for important life history events (i.e., pupping, nursing, and molting) and predictions that climate warming will cause sea ice to decline over the next century (US Federal Register 2012). Given this enhanced regulatory scrutiny, reliable estimates of abundance are needed to balance conservation goals with coastal Alaska Native subsistence needs. Of the four species of ice seals in Alaska, only the bearded seal is harvested near its sustainable limit (Nelson et al. 2019), which is calculated using the best available estimate of abundance. Abundance estimates that are biased low, or are imprecise, can lead to inappropriate restrictions on harvest, while abundance estimates that are biased high, or do not account for all sources of uncertainty, can fail to identify a conservation concern. Thus, reliable estimates of abundance are needed to balance coastal Alaska Native subsistence and conservation needs for the Beringia DPS of bearded seals.

Abundance estimates for bearded seals have previously been based on aerial surveys (e.g., Bengtson et al. 2005; Conn et al. 2014), which are costly, not without risk to survey crews, flown infrequently, and potentially compromised by sources of bias that are difficult to mitigate. Distribution of the Beringia DPS is vast, remote, and extends into Russian waters, requiring international coordination. To date, no survey has covered the entire DPS's range within one continuous year (Young et al. 2023). Combining results from different months in different years and areas is problematic because many bearded seals travel great distances and migrate seasonally (Breed et al. 2018; Cameron et al. 2018; Citta et al. 2018; Olnes et al. 2021). Correcting for seals not seen because they are below the ice or water surface (i.e., availability bias) is also difficult and introduces additional uncertainty and potential for bias in estimates of abundance (London et al. 2024).

Recent developments in close‐kin mark‐recapture (CKMR) methods show great promise for estimating abundance and other demographic parameters for diverse populations and species that are especially difficult to enumerate, such as elasmobranchs, teleost fishes, flying foxes, and boreal caribou (Bravington, Skaug, and Anderson 2016; Hillary et al. 2018; Conn et al. 2020; Trenkel et al. 2022; Delaval et al. 2023; Lloyd‐Jones et al. 2023; Merriell, Manseau, and Wilson 2024), and could likely be applied to marine mammals such as beluga whales, polar bears, and walruses. CKMR methods use individual genotypes as genetic tags to identify kinship relations among pairs of individuals (Skaug 2001). CKMR models incorporate these genetic relationships into population dynamics models by devising case‐specific formulae for the probabilities that particular pairwise comparisons will reveal a specific type of kin, considering details of sampling and biology, and the characteristics of individuals, such as age and sampling date. The probability formulae are based on each individual having a mother and a father, such that a “recapture” is either a direct observation of a parent and its offspring, or an indirect observation in which a pair of siblings, implies the “recapture” of a shared mother or father. The intuitive notion behind CKMR is that, for a given sample size, small populations will yield a higher frequency of observed kin pairs than large populations. However, other parameters such as fecundity‐at‐age and adult survival also affect recapture probabilities. These demographic parameters need to be accounted for when fitting CKMR models and can, in turn, be estimated based on the observed kin pairs.

Modern genotyping methods permit identification of first‐order kin pairs (parent–offspring pairs [POPs] and full sibling pairs [FSPs]) and second‐order kin pairs (half‐sibling pairs [HSPs], grandparent‐grandchild pairs [GGPs], and full thiatic pairs [FTPs, aunt/niece; Table 1]). Early applications of CKMR (Skaug 2001) only used POPs due to the limitations of available genotyping techniques (e.g., microsatellites). Current techniques allow the use of second‐order kin such as HSPs (Bravington, Skaug, and Anderson 2016; Bravington, Grewe, and Davies 2016), which improves precision by increasing the number of kin pairs observed, allows estimation of parameters such as adult survival rates, and can reveal important aspects of a species life history such as reproductive heterogeneity and mating strategy.

**TABLE 1:** Definition of kin relationship acronyms.

Pairwise kinship	Abbrev.	Order of relatedness
Parent–offspring pair	POP	First
Full sibling pair	FSP	First
Half‐sibling pair	HSP	Second
Maternal half‐sibling pair	MHSP	Second
Paternal half‐sibling pair	PHSP	Second
Grandparent‐grandchild pair	GGP	Second
Full thiatic pair (e.g., aunt‐niece)	FTP	Second
Half thiatic pair (e.g., half aunt‐niece)	HTP	Third

Unlike traditional mark‐recapture techniques, CKMR methods are effective even when only dead (e.g., harvested) animals are sampled. Thus, CKMR methods avoid labor intensive and costly research efforts that often result in low sample sizes associated with capturing, marking, releasing, and recapturing marked animals. An important benefit of using harvested animals is the availability of samples that have been routinely collected and archived as part of long‐term harvest monitoring programs, such as that conducted by Alaska Department of Fish and Game (ADF&G) for ice seals. Another advantage of using harvested animals is that the samples, and resulting estimates, may be more representative of the managed population (i.e., the population that is accessible to hunters; Conn et al. 2020) than that sampled by aerial surveys. Aerial surveys are “snap shots” that risk under and over counting when combined across seasons and years. In contrast, the samples we used for CKMR integrate information over seasons and years and result in abundance estimates averaged over these timeframes. Finally, integrating CKMR modeling with a long‐term harvest monitoring program leads to rapid increases in the precision of estimates over time, improving its ability to detect trends in abundance and demographic changes. This is because the expected number of kin pairs increases with the square of the total number of samples rather than linearly as with traditional mark‐recapture techniques.

Biological information is used in CKMR models to estimate abundance and other demographic parameters such as adult survival and fecundity. Bearded seals are long‐lived and not noticeably sexually dimorphic, although females are slightly longer and heavier than males (Burns 1981). It is not uncommon for subsistence harvested individuals to have tooth ages of 20–25 years and ages up to 40 years have been documented (Quakenbush 2020). Peak pupping occurs in late April with the pupping period ranging from mid‐March to early May (Burns 1981). Females have one pup per year, and the pup is weaned within 12–18 days (Burns 1981). Natural mortality rates have been inferred using meta‐analysis (Trukhanova, Conn, and Boveng 2018), as have hunting mortality rates (Nelson et al. 2019) and ages at first ovulation and parturition are known (Crawford, Quakenbush, and Citta 2015; Quakenbush 2020). CKMR models may also be parameterized to estimate factors related to breeding behavior such as the probability of male reproductive success. Long‐term, interannual site fidelity of males to breeding locations has been documented (Van Parijs and Clark 2006), but it is unknown whether bearded seals exhibit mate fidelity. The frequency of FSPs informs this aspect of breeding behavior.

Our objectives were to apply CKMR methods to the Beringia DPS of bearded seals to (1) estimate bearded seal abundance and demographic parameters, (2) gain a better understanding of bearded seal life history, and (3) evaluate the approach for monitoring the status of subsistence harvested marine mammal species and harvested species in general.

## 2 Materials and Methods

### 2.1 Power Analysis

We used a simulation‐based power analysis (Conn et al. 2020) to determine if we had enough samples to achieve parameter estimates with adequate precision, which is primarily determined by the number of kin pairs observed (Bravington, Skaug, and Anderson 2016; Bravington, Grewe, and Davies 2016). Preliminary queries of the ADF&G database indicated paired tissue samples (DNA for genotyping) and jaws (tooth for aging) were available from approximately 1600 bearded seals. We assumed a population abundance of 300,000 seals with known mortality (taken from Trukhanova, Conn, and Boveng 2018) and reproductive schedules (0% maturity until 4 years of age and 100% thereafter). The power analysis predicted four POPs and 20–25 HSPs yielding preliminary models with coefficient of variations (CVs) near 0.2 or up to 0.3–0.4, if accounting for the uncertainty in the survival parameters. We considered these estimates sufficiently precise for the initial phase of a long‐term monitoring program, given that precision will improve, and models will be refined as additional samples are collected and genotyped.

### 2.2 Sample Collection and Preparation

**FIGURE 2:**
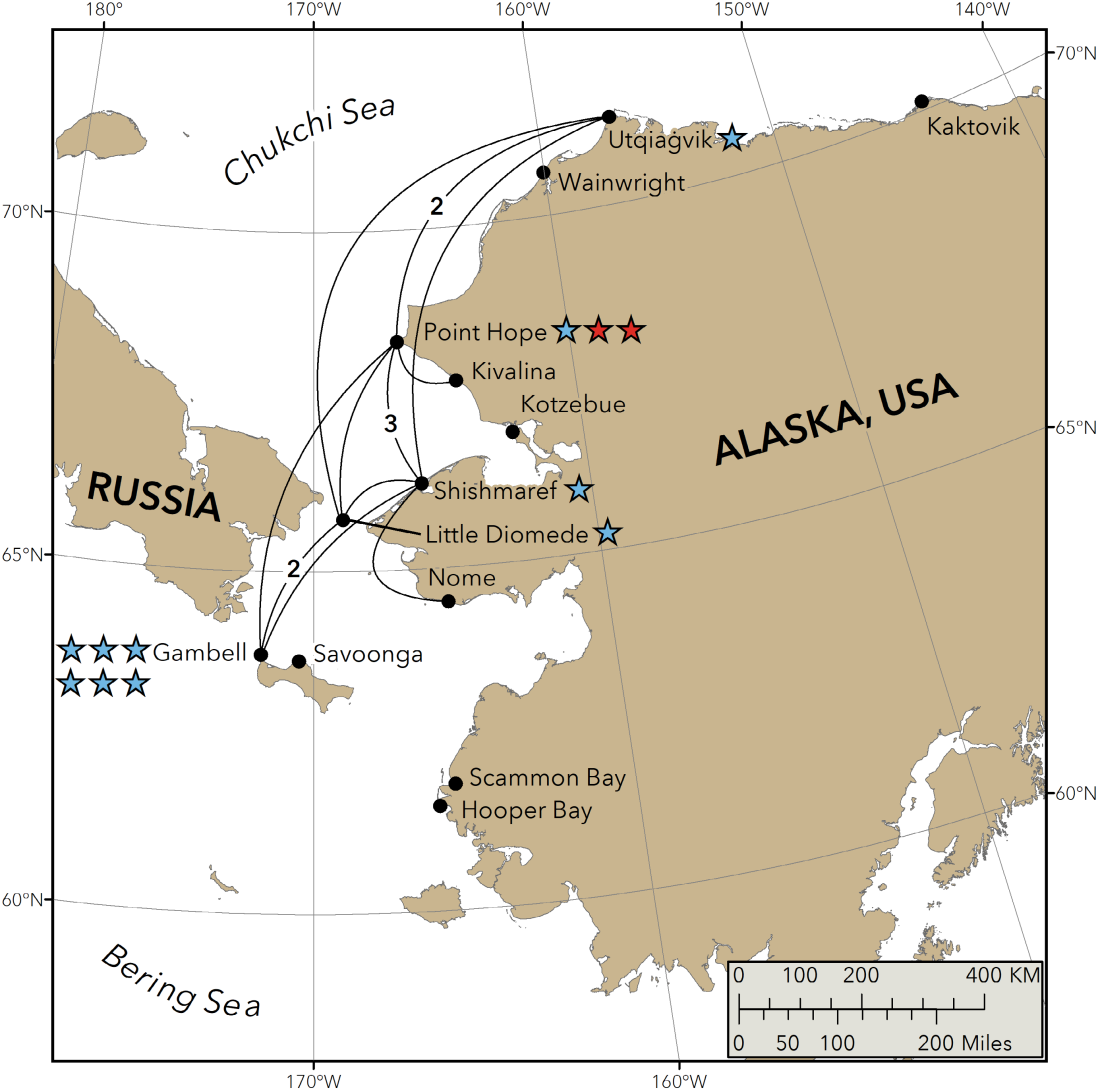
Locations of 13 coastal Alaskan villages spanning the range of bearded seals in the Bering, Chukchi, and Beaufort seas, where samples were collected. The spatial distribution of harvest locations for seals composing our 25 possible HSP‐GGPs and two POPs is also shown with filled stars (POPs red; HSP‐GGPs blue) next to the name of the village indicate the harvest location of both seals in a pair. The seals comprising both POPs were harvested at Point Hope. Curves connect the harvest locations for a pair harvested by different villages. If greater than 1, the number of occurrences of pairs between two villages is shown on the curve. See Table 1 for kin pair acronym definitions.

Samples analyzed for this study were collected from seals harvested by subsistence hunters from 13 Alaskan coastal villages during 1998–2020 (Figure 2) as part of the ADF&G long‐term Ice Seal Biomonitoring Program (Crawford, Quakenbush, and Citta 2015). Samples from two of these villages, Utqiaġvik (formerly Barrow) and Wainwright, were obtained by the North Slope Borough, Department of Wildlife Management (NSB) and incorporated into the ADF&G Ice Seal Biomonitoring Program. Samples were collected by subsistence users, NSB, and ADF&G and processed at ADF&G's Fairbanks Office. Samples not held at ADF&G were archived at the University of Alaska Museum of the North (UAM), and National Oceanic and Atmospheric Administration (NOAA), Southwest Fisheries Science Center. Accession numbers for archived samples are included in Appendix S1. Tissues from 1758 bearded seals (Appendix S1) were subsampled at ADF&G following protocols established by Diversity Arrays Technology Pty Ltd. (DArT) (https://www.diversityarrays.com) and ADF&G's clean sampling procedures implemented (Appendix S2) and sent to DArT for genetic analysis.

Skeletal muscle was the preferred tissue and was analyzed most often (*n* = 1055); however, liver (*n* = 454), skin (*n* = 195), kidney (*n* = 40), spleen (*n* = 7), heart (*n* = 6), and testis (*n* = 1) were also successfully analyzed. Teeth (preferably a lower canine) were sent to Matson's Laboratory, Manhattan, MT, USA, where they were sectioned and aged by counting cementum annuli (see Crawford, Quakenbush, and Citta 2015 for details). Claws were aged in the field or in the laboratory by counting annuli (McLaren 1958; Burns 1969; Burns and Frost 1979) and serve as a secondary source if tooth age was not available. Sex was also determined in the field; however, we used sex determined by genetics (see below) in the models.

### 2.3 Genetic Analysis

DNA extractions, genetic sequencing, and technical quality control were performed by DArT. To develop the DArTcap single nucleotide polymorphism (SNP) panel, DArT genotyped 282 bearded seal tissue samples using the enzyme‐based complexity‐reduction method DArTseq (Jaccoud et al. 2001; Kilian et al. 2012; Grewe et al. 2015; Feutry et al. 2017, 2020). We then selected approximately 4000 SNPs based on technical parameters such as read counts, the location of the SNP along the DNA fragment sequenced (“tag”), and the analysis of duplicate samples (“technical replicates”) to determine high‐quality/low error rate markers (Lloyd‐Jones et al. 2023; [Link], [Link], [Link], [Link], [Link]). One property of the complexity‐reduction approach is that some loci may have heritable null alleles (e.g., due to mutations at primer sites), which are consistently “invisible” (i.e., they are not genotyping errors) and lead to an apparent excess of homozygotes. Although loci with heritable null alleles do somewhat complicate kin finding and QC, they provide some statistical power for identifying kin. We selected SNPs with high power for kin finding by removing SNPs with either low minor allele frequencies or high null frequencies, though loci with modest null frequencies were retained (Hillary et al. 2018; Feutry et al. 2020; Trenkel et al. 2022, and Lloyd‐Jones et al. 2023). Finally, DArT identified seven sex‐linked markers to include in the SNP panel for determining sex genetically. The resulting panel of ~4000 SNPs was then used in targeted genotyping of the remaining 1476 samples using DArTcap (Feutry et al. 2020). Of the 1758 samples submitted for genotyping (282 by DArTseq plus 1476 by DArTcap), 81 (4.6%) were removed by DArT due to poor‐quality DNA, incorrect species (e.g., ringed seal), and duplicates (both planned and inadvertent). The remaining 1677 genotyped samples were considered for kinship evaluation.

Prior to determining kin relationships, we performed additional rigorous quality control (QC) as described by Hillary et al. (2018), Feutry et al. (2020), Trenkel et al. (2022), and Lloyd‐Jones et al. (2023). Initial QC steps included removing DArT's “technical replicate” samples, loci with low read counts (i.e., high chance of genotyping error), loci with low minor allele frequencies (MALF < 0.05), loci with high null allele frequencies (NALF ≥ 0.45), and additional accidental duplicate samples. We then went through an iterative process of (i) removing samples that appeared to have excess or deficient heterozygosity (suggestive of contamination, or of poor‐quality DNA, respectively) or overall atypical multilocus genotype probability, and (ii) removing loci that were out of Hardy–Weinberg equilibrium (HWE) after allowing for potential null alleles and re‐estimating allele frequencies at each step. The HWE filtering step is perhaps the most important and resulted in elimination of about 600 loci (Appendix S3: Figures S3a and S3b). As part of this process, we provisionally re‐introduced samples and loci that had been rejected earlier, to avoid discarding good samples based on bad loci, or vice versa. We carried out this process using QC functions in the R package “Kinference” (Bravington, Miller, and Baylis 2021). We retained 2569 loci, all of which were consistent with HWE and general predictions of genotype distribution and retained genotypes for 1484 seals providing for approximately 1 million pairwise comparisons for each kin type evaluated. Taking a strict approach to QC before performing the pairwise comparisons safeguarded against making erroneous kin determinations. The ultimate test of the QC process is whether the kin finding results agree with Hardy‐Weinberg theoretical distributions, which they do (Appendix S3: Figures S3c–S3e). The genotypes for the 1484 seals used in the models are permanently and publicly archived in the DRYAD Digital Repository.

Mitochondrial DNA (mtDNA) haplotype sequences are required to distinguish between paternal (PHSP) and maternal (MHSP) half‐sibling pairs. The mtDNA haplotype sequences of samples comprising HSPs were generated using a targeted genotyping assay (DArTag, https://www.diversityarrays.com/services/targeted‐genotying/) developed by DArT. DArTags were designed to re‐sequence short but overlapping regions covering the mtDNA control region. The targeted regions were amplified, and sample‐specific barcodes were attached. The DArTag products were then sequenced on an HiSeq 2500 (Illumina, San Diego, CA, USA), demultiplexed, and aligned to the published bearded seal mitogenome (GenBank AM181027.1). DArT‐generated trimmed and aligned sequences (GenBank PP178967.1 to PP179021.1) were used to determine co‐inherited mtDNA (i.e., to help distinguish PHSPs from MHSPs).

### 2.4 Kinship Inference

To conduct kinship inference, we used the likelihood‐based approach developed by Bravington, Skaug, and Anderson (2016) and Bravington, Grewe, and Davies (2016) for calculating likelihood‐ratio kin identification statistics. Briefly, this approach relies on calculating pseudo‐log odds (PLOD) scores for the frequency of co‐inherited DNA associated with different kinship types (e.g., POP, FSP, HSP, and GGP, and unrelated pair [UP]) as a way of discriminating between the different kinship groups. Given the large quantity of loci examined, POPs and FSPs could be reliably discriminated but there was some risk of mistaking second‐order kin (e.g., HSP or GGP) for third‐order kin (a false‐negative error) or mistaking third‐order kin for second‐order kin (a false positive error). To mitigate the latter, we imposed a lower threshold for second‐order kin PLOD scores, estimated the associated false‐negative rates and incorporated them into kinship probability formulae. For further information on kinship inference, see Appendix S3.

### 2.5 Statistical Modeling of Kinship Relationships

#### 2.5.1 Demographic Modeling

Underpinning all our CKMR analyses is an age‐structured population dynamics model composed of annual abundance, annual survival probabilities, and fecundity parameters. We assumed a post breeding census, in which the number of new recruits each year is given by:

$$N_{t,0}^{F}=N_{t,0}^{M}=0.5\sum_{a=1}^{39}N_{t-1,a}^{F}\varphi_{a}f_{a}$$

where $N_{t,a}^{F}$ gives the number of females in age class *a* (males use the superscript “*M*”) alive at time *t*, *a =* 0 corresponds to pups, *φ*_*a*_ is the probability of survival from age class *a* to *a* + 1, and *f*_*a*_ is the fecundity, or average number of offspring born to a female in age class *a*. Note that we assume a 50/50 sex ratio at birth, which is a reasonable assumption given data collected on pups in the field (Fedoseev 2000). Later age classes are propagated forward as a function of age‐specific survival (i.e., $N_{t,a}^{F}=N_{t,a}^{M}=0.5N_{t-1,a-1}\varphi_{a-1}$ for *a* > 0, *t* > 0). During the initial year of the population dynamics model (*t* = 1), we set abundance values equal to stable stage proportions from the associated matrix population model (Caswell 2001). We started our population model one maximum lifespan (40 years) prior to data collection to allow us to model prior reproduction of parents.

To provide our model with information on survival‐at‐age, we adopted an informative Bayesian prior distribution based on a hierarchical meta‐analysis of phocid seal mortality (Trukhanova, Conn, and Boveng 2018). This meta‐analysis used a reduced additive Weibull distribution (RAW; Choquet et al. 2011) to model natural mortality as a function of age for different phocid seal species and populations. The RAW model is characterized by a “bathtub” shaped curve for mortality (i.e., high mortality at young ages, low mortality for young adults, and increasing mortality for the oldest individuals). According to this framework, age‐specific annual survival at age *a* $\left(\varphi_{a}\right)$ is given by:

$$\varphi_{a}=\exp\left(-{\left(\eta_{1}a\right)}^{\eta_{2}}-{\left(\eta_{1}a\right)}^{\frac{1}{\eta_{2}}}-\eta_{3}a\right)$$

where *η*_1_, *η*_2_, and *η*_3_ are estimated parameters. The values of these parameters from hierarchical analysis (Trukhanova, Conn, and Boveng 2018) were *η*_1_ = 0.055, *η*_2_ = 2.80, and *η*_3_ = 0.076 (Conn et al. 2020). We did not include hunting mortality because harvest rates are less than 3% based on estimates of harvest and abundance (Nelson et al. 2019), and because the CKMR model ultimately allowed for flexibility to adjust survival downward to reflect our assumption of population stability (see below).

We set fecundity‐at‐age values equal to those reported by Conn and Trukhanova (2022), who fitted generalized additive models to data from seals collected in the Bering and Chukchi seas. These data represented the proportion of age *a* females who had given birth or were pregnant in the spring. Seals were from Alaska Native subsistence harvests collected by ADF&G and data from Russia in the 1980s (Fedoseev 2000).

**FIGURE 3:**
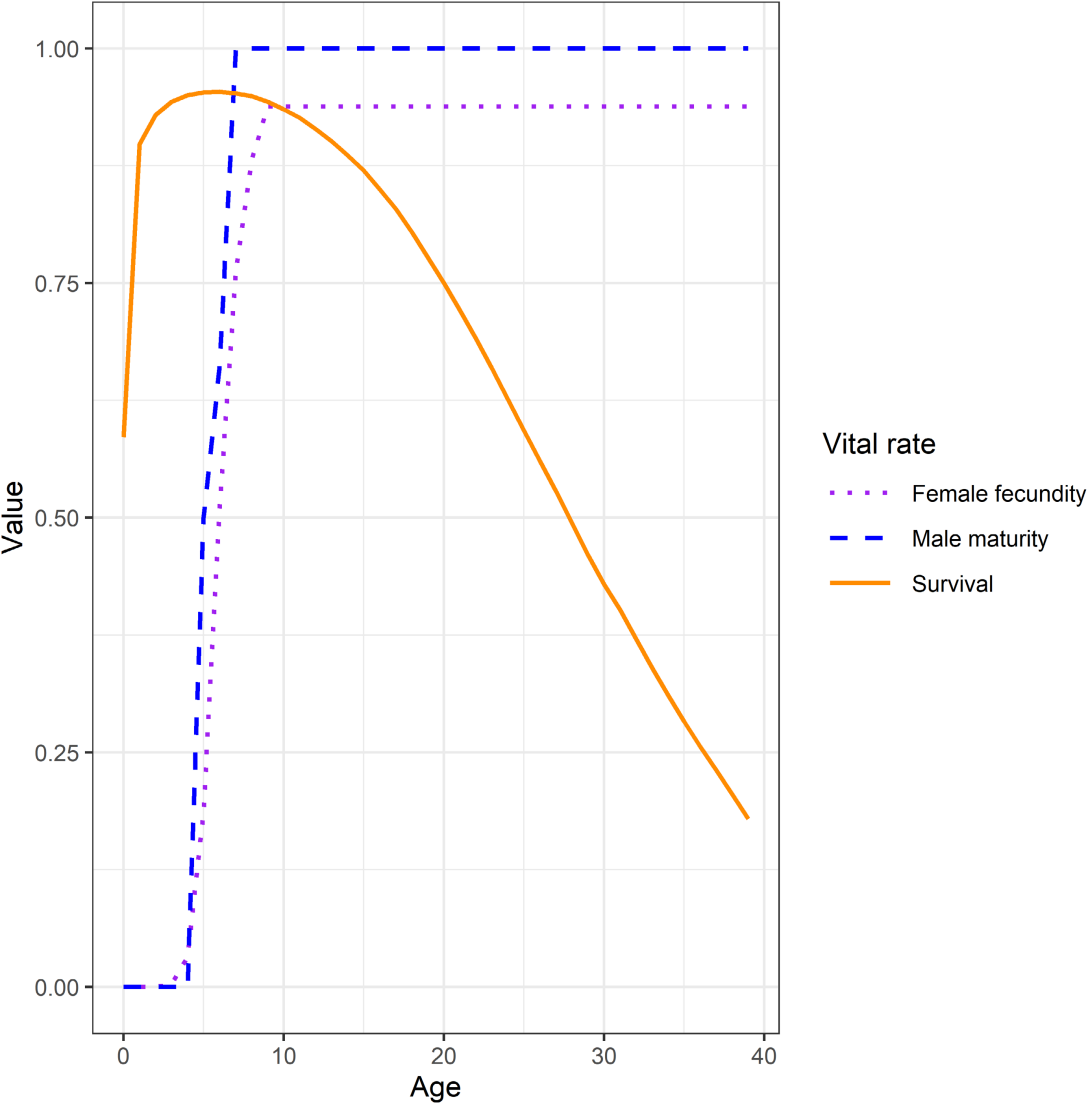
Age‐specific values of bearded seal female fecundity, male maturity, and survival values used as inputs to CKMR models. Fecundity and maturity schedules were fixed during estimation, while parameters of the survival function were estimated (the survival schedule shown is the expectation from the Bayesian prior).

CKMR paternal kinship probabilities (including PHSPs) rely on relative paternal reproductive output as a function of age. We based these calculations in part on male maturity reported by Conn and Trukhanova (2022), which were derived from collections from the Bering and Okhotsk seas (Tikhomirov 1966). Survival, female fecundity, and male maturity‐at‐age (*m*_*a*_) are plotted in Figure 3.

The combination of our set fecundity‐at‐age values and survival‐at‐age Bayesian prior yielded a finite rate of population increase of *λ* = 1.04 (4% increase per annum). This was clearly undesirable, because we did not want to presuppose such an increase before we started analyzing CKMR data. Therefore, we let the CKMR model estimate updated RAW parameter values, subject to a constraint enforcing *λ* ≈ 1.0. This procedure had the advantage that CKMR data could help inform where the survival curve should be adjusted to best meet the *λ* ≈ 1.0 constraint. It also resulted in increased mortality, which was desirable because prior survival parameters did not incorporate hunting mortality.

#### 2.5.2 The HSP‐GGP‐POP CKMR Model

We developed an HSP‐GGP‐POP CKMR model, in part because our power analysis revealed that there would not be enough POPs to be able to rely on POPs alone (Hillary et al. 2018). However, given the age distribution of the sampled individuals, and the spread in sampling years, we could not simply assume that all the second‐order kin would be HSPs; therefore, we needed to consider the possible presence of GGPs (see Bradford et al. 2018 for a similar situation involving grey nurse sharks). As described in Appendix S3, we were able to rule out the presence of FTPs, the third possible type of second‐order kin, due to the lack of FSPs. GGPs have the same expected proportions of co‐inherited nuclear DNA as HSPs so the two cannot be distinguished by genetics alone (at least not without a far larger SNP panel and genomic analysis; see Ramstetter et al. 2017). Distinguishing features of GGPs are at least one of the pair must be an adult (assuming lethal sampling) and the birth gap must be at least twice the age of maturity. Therefore, ages and birth gaps for seal pairs were used to assess the relative likelihood of HSPs versus GGPs.

We based statistical inference on the joint pseudo‐likelihood:

$$L=L_{\mathrm{pop}}L_{\mathrm{hsp}/\mathrm{ggp}}f\left(\eta \right)\Lambda_{\lambda }$$

where *L*_pop_ is a joint likelihood for POPs, *L*_hsp/ggp_ is a joint likelihood for HSPs and GGPs, *f*(*η*) are penalties on RAW survival parameters if they deviate from their prior mean (essentially, taking the place of a prior distribution), and Λ_*λ*_ is a penalty on *λ* values deviating from *λ*_0_, which is set by the analyst (set to 1.0 for most models). The likelihoods *L*_pop_ and *L*_hsp/ggp_ were product Bernoulli, with probabilities $p_{\mathrm{ij}1}$ and $p_{\mathrm{ij}2}$, respectively, where $p_{\mathrm{ij}1}=\mathrm{Pr}\left(\mathrm{POP}|x_{i},x_{j}\right)$ and $p_{\mathrm{ij}2}=\mathrm{Pr}\left(\mathrm{HSP}|x_{i},x_{j}\right)+\mathrm{Pr}\left(\mathrm{GGP}|x_{i},x_{j}\right)$. Here, $x_{i}\;\text{and}\;x_{j}$ are covariate vectors associated with animal *i* and animal *j* (including age, sex, and mitochondrial haplotype). To account for GGPs, we modeled apparent HSPs as a mixture of HSPs and GGPs (HSP‐GGPs). The probability of a GGP differs depending on whether the two animals being compared have the same mtDNA haplotype (Appendix S4: Figure S4a). For instance, if the older animal is female, there is about a 50% chance that GGPs will share mtDNA (depending on whether the unobserved parent is female); conversely, if the older animal is male, the chance of sharing mtDNA is zero (neglecting haplotypes shared by chance). Similar probability formulae form the basis of any CKMR application, but they were quite complicated in this case. Detailed descriptions as well as information about prior distributions and penalties on population growth rate are provided in Appendix S4.

To fit this model, we programmed the log pseudo‐likelihood (LPL) in Template Model Builder (TMB; Kristensen et al. 2015). Conditioning on observed kinship observations (i.e., the *y*_*ijk*_), we then minimized the negative LPL as a function of bearded seal abundance and survival parameters using the “nlminb” function in R (R Core Team 2022). Because many of the pairwise kinship probabilities are the same (e.g., for individuals of the same sex and with the same birth years and year of death), some computational efficiency is gained by grouping prospective kin pairs by common covariate values (e.g., birth dates, sex of parent). The code and data to implement our analysis are permanently and publicly archived in the DRYAD Digital Repository.

#### 2.5.3 Heterogeneity in Male Breeding Success

Our initial HSP‐GGP‐POP model assumed adult males and females contributed equally to the genetics of the offspring. However, male bearded seals are known to maintain underwater territories during breeding, and thus, there may be male competition for mates (Van Parijs, Lydersen, and Kovacs 2003). Such competition can result in persistent heterogeneity in adult male reproductive success (e.g., if older or higher quality males are able to breed with more females than younger or lower quality males). Furthermore, male reproductive schedules were based primarily on the physiological ability to breed (e.g., presence of viable sperm) rather than observed reproductive behavior, and thus may overestimate the fraction of reproducing males in the population. To address this issue, we developed an alternative CKMR model that estimated the breeding fraction (*π*) of the total number of sexually mature males (see Appendix S4). As we shall show (in Section 3), this male heterogeneity model fit sex‐specific HSP‐GGP counts better than a model where π was fixed to 1.0; therefore, we designated it as the base model for examining the effects of alternative HSP/UP PLOD thresholds and various trend scenarios.

#### 2.5.4 Alternative Trend Scenarios

Another possible source of structural uncertainty is our assumption that abundance is constant over time. Therefore, we investigated four trend scenarios, corresponding to *λ*_0_ values of 0.96, 0.98, 1.02, and 1.04. We compare joint pseudo‐likelihood functions for each of these scenarios to judge their relative support. For this exercise, we limit comparison to the joint kin‐pair likelihood, *L*_pop_ *L*_hsp/ggp_ (i.e., omitting penalty contributions).

## 3 Results

### 3.1 Sample Characteristics

Associated information for the 1484 genotyped seals used in the models including location of harvest, year of harvest, date of harvest, age at harvest, tooth or claw age, tooth age quality, type of tissue analyzed for genetics, the archiving laboratory, and the genetically determined sex is permanently and publicly archived in the DRYAD Digital Repository. Briefly, the number of samples per year increased from < 10 in the late 1990's to 80–90 by 2009 and averaged just under 100 per year through 2020. Although ages range up to 34 years, the age distribution is strongly skewed toward younger seals with pups comprising 46.0% (683 of 1484) of the samples and pups plus juveniles ≤ 3 years old comprising 65.3% (969 of 1484). Birth years ranged from 1976 to 2020. The seals are evenly distributed by sex with 751 females and 733 males. Although seals were harvested at 13 communities, five communities provided 95.8% of the samples: Gambell (*n* = 483) and Diomede (*n* = 161) in the Bering Sea and Shishmaref (*n* = 310), Point Hope (*n* = 315), and Utqiaġvik (*n* = 153) on the Chukchi Sea coast (Figure 2).

Of these samples, 1412 (95%) were aged using teeth and 62 were aged using claws. Ten seals were not aged and, therefore, not used in the CKMR models. Of the 62 aged by claws, 57 (92%) were pups. Claw ages have been shown to be reliable for pups when compared to paired tooth ages (ADF&G, unpublished data). The other five claw ages used were between 7 and 10 years. Claw ages for these older animals are considered minimum ages due to wear of the annuli (McLaren 1958; Burns 1981). Matson's laboratory assigned 76.6% of the tooth ages (1081 of 1412) the highest reliability code A (i.e., the cementum characteristics of the tooth section matched those of the standardized cementum aging model for that species and tooth type), 16.6% were assigned code B (i.e., correct age is expected to be within the age range given), 0.8% were assigned code C (i.e., error is likely and may occur within the age range given), and 6.0% were not assigned a code. A single age was assigned to 976 (69%) teeth, an age range of 1 year was assigned to 210 (15%) teeth, and an age range of 2 years was assigned to 136 (10%) teeth. Age ranges greater than 2 years were assigned to 90 (6%) teeth. For CKMR analyses in this paper, we treated assigned ages as if they were exact.

### 3.2 Kinship Determinations

Demographic and harvest data indicated that nine of the 11 POPs we identified (Appendix S3) were mother/pup pairs. All nine pairs were comprised of an adult female and pup that were harvested in the same month and year at the same location, including six pairs known to be harvested on the same day. Because mother/pup pairs represent dependent captures, they cannot be used for CKMR modeling. Thus, we included only two POPs in the models and omitted all parent–offspring kinship comparisons where samples were obtained in the same year.

**FIGURE 4:**
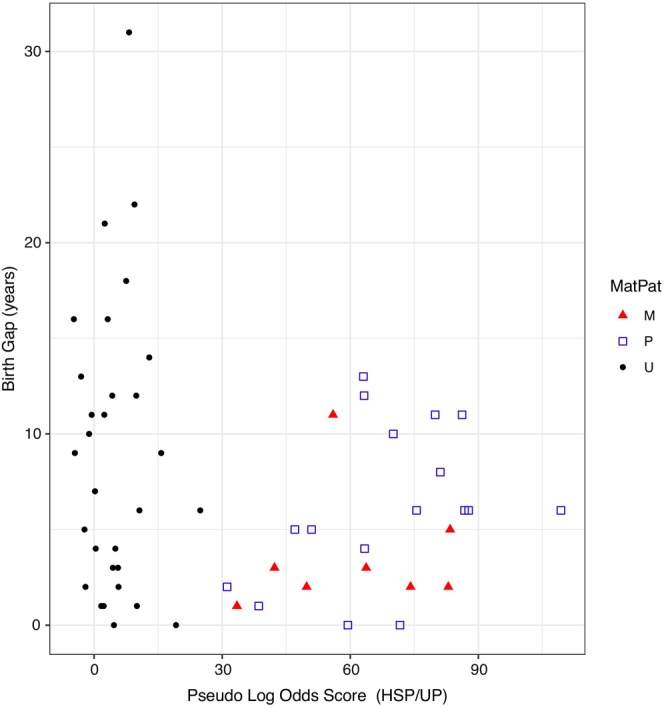
Birth gap by HSP/UP PLOD values for HSP‐GGPs and third‐order kin. We performed mtDNA analysis on pairs with HSP/UP PLOD scores > 30 (i.e., potential HSP‐GGPs) to identify those that share mtDNA (maternally related, M) and those that do not (paternally related, P). Whether likely third‐order kin (i.e., pairs with PLOD scores < 30) are maternally or paternally related is unknown (U).

The number of HSP‐GGPs we identified depended on which of the three values of PLOD thresholds we used to limit false positive errors (Appendix S3). Specifically, PLOD thresholds of 30, 40, and 50 resulted in 25, 22, and 19 HSP‐GGPs, and estimated false‐negative probabilities of 0.0002, 0.003, and 0.02, respectively. All four potential GGPs have PLOD scores > 50 (i.e., 56, 63, 80, and 86) thus were retained in all models. We examined demographic data to help guide our choice of the threshold to use for our base models. The lack of FSPs led to half thiatic pairs (HTPs, e.g., half aunt‐niece) being the only third‐order kin of concern. HTPs typically have birth gaps with a mode near one generation; although possible, near‐cohort‐HTPs are rare. Our potential HSP‐GGPs with the lowest PLOD scores (< 47) had birth gaps much less than a generation (Figure 4) providing some justification for a threshold around 30. However, selecting a lower threshold increases the risk of false positives, which we wanted to avoid. As a result, we used the middle PLOD threshold value of 40 to determine the number of HSP‐GGPs and associated false‐negative rate in all statistical models except for the two sensitivity runs specifically designed to assess PLOD threshold impact.

Mitochondrial DNA analysis of our 25 potential HSP‐GGPs indicated that 17 (68%) do not share mtDNA while eight (32%) do. Because most of these pairs were HSPs, it suggested a higher proportion of paternally related HSPs (i.e., which do not share mtDNA) than maternally related HSPs. This is a departure from the expected ≈50:50 ratio for HSPs when all adult males and females are equally likely to breed. For example, if we assume that all kin pairs are HSPs, a binomial CDF test with equal probability of MHSPs and PHSPs gives a *p*‐value of 0.05 (below, we refine this test to account for the possibility of GGPs based on model results).

Covariate values for our 27 possible kin pairs (POPs and HSP‐GGPs) are provided in Appendix S5. Briefly, the year of harvest ranged from 2002 to 2020, birth years ranged from 1993 to 2020, and ages ranged from 0 to 14. Most (52 of 54) seals had tooth ages and two pups had claw ages. Most of the teeth (43 of 52, 82.7%) had the highest tooth age reliability code A, seven teeth had code B, one tooth had code C, and one tooth had no quality code. Four seals were assigned age ranges of 1 year, and seven seals were assigned age ranges of 2 years. Using the covariate data, we identified two PHSPs that were likely from the same birth year consistent with males breeding with multiple females in the same year. We also identified three instances indicative of aging error. The harvest year of the parents of the two POPs preceded the birth year of the offspring, indicating that the age of the offspring was underestimated. To rectify these discrepancies in our CKMR analysis, we increased the age of these two offspring by 1 year. Furthermore, we identified one same‐cohort MHSP, which is not possible because bearded seal females have only one pup per year. To rectify this, we increased the age of the oldest seal from 6 to 7 to create a birth gap of 1 year. The other seal was harvested as a pup; thus, we considered its age exact. These age discrepancies demonstrate the eventual need to develop a model that accommodates aging error when sample sizes are sufficient and additional data on aging error are available.

### 3.3 Spatial Distribution of Kin Pairs

As we would expect, most (52 of 54) samples making up the 27 kin pairs were harvested by the top five contributing villages. Twelve of the 27 kin pairs (44%) were harvested by the same community, six in Gambell, three in Point Hope (the two communities with the largest contribution of samples), and one each in Utqiaġvik, Shishmaref, and Little Diomede. However, a greater percentage (15 of 27, 56%) of kin pairs were harvested in different communities showing a great deal of spatial mixing (Figure 2).

### 3.4 Statistical Modeling of Kinship Relationships

#### 3.4.1 HSP‐GGP‐POP CKMR Models

As described above, we initially ran the *HSP‐GGP‐POP* models using the intermediate threshold HSP/UP PLOD score of 40, which included 22 of the 25 potential HSP‐GGPs identified by kinship inference. The abundance estimate was $\hat{N}$ = 2.32 × 10^5^ (Table 2, Model 1). Owing to the strong constraint on *λ* (i.e., = 1.0), the estimate is fairly (and perhaps overly) precise with CV = 0.21. By way of comparison, an HSP‐POP model (i.e., assuming 22 HSPs and no GGPs) provided a lower estimate of $\hat{N}$ = 1.85 × 10^5^ CV = 0.21. The lower abundance estimate was expected because this model does not consider that some of the HSPs have some chance of being GGPs, thereby inflating the number of HSPs and decreasing abundance. As we anticipated from age and birth gaps, there were four pairs with potential to be GGPs, though their relative probabilities still favor them being HSPs (Table 3). The greatest probability of being a GGP is 0.43 for one pair; the other three have probabilities ≤ 0.20.

**TABLE 2:** CKMR estimates of abundance with CV for the HSP‐GGP‐POP models.

Model	FSP/UP PLOD threshold	*λ*	Male breeding heterogeneity	# HSPs & GGPs	Pat/Mat	$\hat{N}$	CV
1	40	1.0	N	22	15/7	2.32 × 10^5^	0.21
2	40	1.0	Y	22	15/7	4.09 × 10^5^	0.35
3	30	1.0	Y	25	17/8	3.65 × 10^5^	0.33
4	50	1.0	Y	19	14/5	5.35 × 10^5^	0.41
5	40	0.96	Y	22	15/7	4.37 × 10^5^	0.35
6	40	0.98	Y	22	15/7	4.12 × 10^5^	0.35
7	40	1.02	Y	22	15/7	4.28 × 10^5^	0.35
8	40	1.04	Y	22	15/7	4.70 × 10^5^	0.35

*Note:* HSP‐GGP‐POP models were run using three different HSP/UP PLOD score thresholds and five trends (*λ*, lambda values), one being constant abundance (*λ* = 1.0). Varying the threshold resulted in models with varying numbers of HSP‐GGPs and varying ratios of paternal (Pat) to maternal (Mat) related pairs. However, all models included the four possible GGPs. The first two models were run with a PLOD threshold of 40 both with and without accounting for male breeding heterogeneity. We selected Model 2, which accounts for heterogeneity, as the base model for varying threshold scores and trend in Models 3–8.

**TABLE 3:** Relative probabilities of individual seals being HSPs vs. GGPs given by the base model.

PLOD value	Birth yr *i*	Age *i*	Birth yr *j*	Birth gap	mtDNA	Rel_prob_HSP
42	2008	0	2011	3	M	1.00
47	2006	2	2011	5	P	1.00
50	2015	0	2017	2	M	1.00
51	2004	0	2009	5	P	1.00
**56**	**1997**	**7**	**2008**	**11**	**M**	**0.80**
59	2015	0	2015	0	P	1.00
63	2007	0	2020	13	P	1.00
**63**	**1993**	**13**	**2005**	**12**	**P**	**0.57**
63	2004	4	2008	4	P	1.00
64	2012	1	2015	3	M	1.00
70	2002	0	2012	10	P	1.00
72	2010	1	2010	0	P	1.00
74	2009	1	2011	2	M	1.00
76	2012	1	2019	6	P	1.00
**80**	**1999**	**6**	**2010**	**11**	**P**	**0.90**
81	2009	0	2017	8	P	1.00
83	2012	0	2014	2	M	1.00
83	2004	0	2009	5	M	1.00
**86**	**1998**	**14**	**2009**	**11**	**P**	**0.90**
87	1999	12	2005	6	P	1.00
88	2006	0	2012	6	P	1.00
109	2008	4	2014	6	P	1.00

*Note:* Also shown are HSP/UP PLOD values, birth gaps, and whether maternally (M) or paternally (P) related. Possible GGPs are in bold. The oldest seal in each pair is depicted as *i* the younger as *j*. The relative probability of being an HSP vs. GGP (Rel_prob_HSP) is calculated as $\frac{\mathrm{Pr}\left(\mathrm{HSP}|x_{i},x_{j}\right)}{\mathrm{Pr}\left(\mathrm{GGP}|x_{i},x_{j}\right)+\mathrm{Pr}\left(\mathrm{HSP}|x_{i},x_{j}\right)}$.

Under the PLOD 40 threshold, there were seven MHSP‐GGPs compared to 15 PHSP‐GGPs (Table 2). By contrast, conditional on estimated parameters, the number of expected MHSP‐GGPs was 9.1 and the number of expected PHSP‐GGPs was 11.3. A chi‐square test with expected and observed numbers of HSPs by sex only, generated a *p*‐value of 0.17 ($\chi^{2}$ = 1.9, df = 1); however, with only one degree of freedom and low sample sizes we did not have much power to detect an effect. An alternative explanation (other than random chance) for the difference in paternal vs. maternal HSP‐GGP counts is that there are fewer successful male breeders.

#### 3.4.2 Male Breeding Heterogeneity

We also used the HSP‐GGP‐POP model with the threshold at PLOD = 40 to investigate the effect of persistent heterogeneity in an adult male breeding success scenario. Running a model that drew upon information in the ratio of paternal vs. maternal HSP‐GGPs to account for heterogeneity in male reproductive success resulted in $\hat{\pi }=0.34$ (standard error [SE] = 0.15), consistent with, a relatively small fraction (34%) of reproductively mature males being regularly (i.e., over time) successful at producing offspring. Adjusting for male reproductive heterogeneity led to a substantial increase in estimated abundance, which became $\hat{N}=4.09\times 10^{5}$, CV = 0.35 (Table 2, Model 2). Using AIC to compare this model to the same model with π fixed to 1.0 indicated that the former fit the sex‐specific HSP‐GGP counts better than the latter (ΔAIC = 4.3). Expected MHSP‐GGP and PHSP‐GGP counts were also much closer to observed values (5.15 and 15.14, respectively). For these reasons, we selected the model accounting for heterogeneity in the breeding success of adult males as the base model for examining the effect of alternative UP/HSP PLOD thresholds and various trend scenarios.

#### 3.4.3 Alternative Thresholds for HSP‐GGP Inclusion

Looking at estimates from different model runs in which we vary the threshold and false‐negative HSP‐GGP probabilities, we see that abundance estimates increased with increasing PLOD threshold value. The PLOD 30 threshold resulted in $\hat{N}$ = 3.65 × 10^5^ and the PLOD 50 threshold an $\hat{N}$ = 5.35 × 10^5^ (Table 2, Models 3 and 4, respectively). In principle, the choice of threshold should not affect the abundance estimates very much (unless it is set too low) because the loss of true kin pairs with a higher threshold should be automatically compensated for by including the estimated false‐negative probability in the model. However, that argument relies on the false‐negative probability being accurately estimated. In our case, the variance estimate derived from the upper 50% of the HSP PLOD histogram (Figure S3e) suggested that the false‐negative probability was close to zero for each of the PLOD thresholds even though increasing the threshold from 30 to 50 dropped three HSPs. Our view is that the variance of the HSP PLOD distribution (the curve in Figure S3e) was likely underestimated due to a relatively low number of samples above the HSP mean (currently 8). As more samples are collected and analyzed, we suspect that the estimate of this variance will become more accurate, resulting in higher false‐negative probabilities and greater similarity in abundance estimates for models with different PLOD thresholds.

#### 3.4.4 Alternative Population Trend Scenarios

We investigated alternative population trend scenarios for HSP‐GGP‐POP models with a threshold at PLOD = 40 (Table 2, Models 5–8). For the increasing and decreasing population scenarios, we reported average estimated abundance ($\hat{N}$) from 1990 to 2020 in Table 2. Log LPL values were similar for the five trend (*λ*) values, with *L* = −302.07, −300.36, −299.98, −301.45, and −305.65 for *λ* = 0.96, 0.98, 1.0, 1.02, and 1.04, respectively. Our CKMR data do not appear to carry precise information about trend yet, at least with current sample sizes, though they could be used to rule out the possibility of large increasing (*λ* = 1.04) and large decreasing (*λ* = 0.96) trends.

#### 3.4.5 Survival Estimates

**FIGURE 5:**
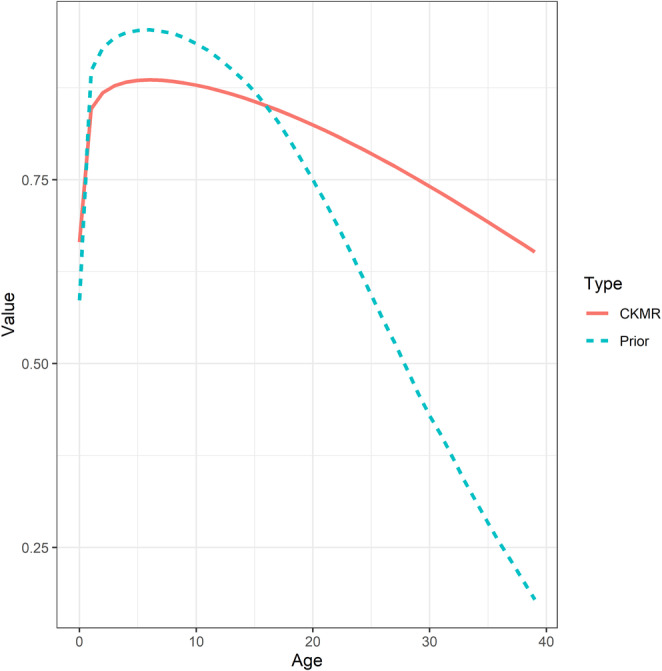
Prior and posterior estimates of survival‐at‐age, the later corresponding to the base model, which accounts for heterogeneity in the breeding success of adult males (Table 1; Model 2).

Our CKMR analysis also provided information on survival of breeding adults as depicted by the Bayesian posterior distribution peaking at a lower value but with higher survival at older ages than the prior distribution (Figure 5). These estimates of average adult survival reflect information contained in birth gaps of HSPs because the adult responsible for each HSP needs to live long enough to generate the observed birth gap (Bravington, Skaug, and Anderson 2016; Bravington, Grewe, and Davies 2016). However, our estimation procedure also influenced these estimates of survival because we were only able to achieve a constant population growth rate by adjusting survival‐at‐age parameters (*η*_1_, *η*_2_, and *η*_3_).

Examining the birth gaps directly (i.e., independent of the model) suggested that paternally related HSP‐GGPs had larger birth gaps on average than maternally related HSP‐GGPs (Figure 4; 6.2 vs. 3.3 years; *p*‐value = 0.13). That the paternally related HSP‐GGPs had larger birth gaps implied that adult male survival, on average, was greater than that of adult females. This preliminary finding was inconsistent with the current understanding of no difference between adult male and female survival (Trukhanova, Conn, and Boveng 2018).

## 4 Discussion

In this first successful application of CKMR to estimate the abundance of a marine mammal, we demonstrate how CKMR methods can be used, in conjunction with a biomonitoring program, to assess and monitor the abundance and demographic parameters of a harvested species. An analysis of kinship using 1484 harvested bearded seals that were genotyped, sexed, and aged resulted in two POPs and 25 potential second‐order kin pairs, mostly HSPs. No FSPs were found, indicating a lack of mate fidelity. An analysis of the mtDNA of the second‐order kin pairs showed that most were paternally related, consistent with a relatively small percentage (34%) of males regularly producing offspring over time.

Modifying the CKMR base model to account for the disparity in male reproductive success substantially increased the abundance estimate from ~232,000 to ~409,000, which is of similar magnitude to aerial survey estimates. For instance, recent surveys in the Bering, Chukchi, and Beaufort seas estimate the Beringia DPS at around 500,000 bearded seals (P. Boveng, personal communications, April 2024). If bearded seals counted in the western region of the aerial survey areas do not mix with bearded seals in the eastern region such that they are not available for harvest by Alaska Native subsistence hunters, then the CKMR population estimates should be smaller than the estimate for the entire Beringia DPS. Under such circumstances, however, the CKMR abundance estimate would be more relevant for management purposes in Alaska. However, movement studies of juvenile bearded seals tagged with satellite transmitters along the Alaska coast show bearded seals travel great distances including into Russian waters although they spend more time on the Alaskan side of the Bering and Chukchi seas than on the Russian side (Citta et al. 2018; Breed et al. 2018; Olnes et al. 2020, 2021). If adults exhibit similar wide‐ranging movement patterns, then we would expect the two methods to yield similar estimates of abundance.

In addition to estimates of abundance, the CKMR models also produced estimates of average adult survival (Figure 5). The posterior distribution diverged from the Bayesian prior distribution developed from meta‐analysis (Figure 3; Trukhanova, Conn, and Boveng 2018), in part, due to information in the birth gaps of the HSPs (Figure 4). Unfortunately, because our estimation procedure also influenced these survival estimates we are disinclined to provide a biological interpretation until we obtain sample sizes sufficient to estimate population trend directly, which would result in survival inferences unaffected by trend constraints. However, the observed birth gaps are not model dependent and those for PHSPs are, on average, greater than those of MHSP (Figure 4). This implies the survival of breeding males is greater than that of breeding females. We did not predict sex‐specific adult survival using historical demographic data and given the limited number of kin pairs, we are reluctant to interpret it as such. Instead, we consider it an interesting finding that warrants further evaluation as sample sizes increase.

This study greatly improved poorly understood aspects of bearded seal life history, particularly their mating strategy. Even though the number of kin pairs is relatively small, the absence of FSPs (compared to ~20 HSPs) suggests that bearded seals do not exhibit interannual mate fidelity. This was unexpected because individual males are known to maintain the same breeding territories for many years (Van Parijs and Clark 2006) and females could locate and mate with the same male in subsequent years producing FSPs. On the other hand, we found two examples of same birth year PHSPs indicative of males breeding with multiple females in the same year, confirming the expectation of polygyny given male breeding behavior (Van Parijs and Clark 2006). Even more unexpected was the evidence for, and degree of, heterogeneity in male breeding success manifested in the dominance of PHSPs over MHSPs. The possibility that, on average, only one third of the adult males successfully produce offspring over time has important implications for effective population size, evolutionary potential, population dynamics, and abundance estimation. That said, we recognize the uncertainty in the estimated proportion is relatively large, $\hat{\pi }=0.34$ (SE = 0.15); thus, additional data are required to confirm this finding. Interestingly, our estimate of annual adult male breeding success is the same as the estimated proportion of adult males maintaining stationary breeding territories (34%) relative to males that roam (66%) off the coast of Point Barrow, Alaska (Van Parijs and Clark 2006). If fewer males have stationary breeding territories, but those males are more successful breeders than those that roam, then this behavior could explain the heterogeneity found in male reproductive success in this study. However, a viable alternative is that the males do not start breeding until a later age than indicated by our maturity‐at‐age function (Figure 3). By experimenting with a variety of male maturity curves, we found that male sexual maturity would need to be delayed by 8 years beyond what we used to result in $\hat{\pi }=1.0$. Differences in physiological vs. social maturity are known for male pinnipeds such as walruses (*Odobenus rosmarus*, Fay 1982) and northern fur seals (*Callorhinus ursinus*, Gentry 1997). Walruses also establish breeding sites (leks) where they produce sounds (clicks, knocks, and bell‐like or gong sounds) to advertise for females (Ray and Watkins 1975). Male walruses are sexually mature 5 to 6 years before they can physically compete with other males to successfully breed (Fay 1982).

Our study is in the early phase of a long‐term project and includes fewer kin pairs than the 50–100 recommended for robust application of CKMR by Bravington, Skaug, and Anderson (2016) and Bravington, Grewe, and Davies (2016). Limited kin pairs had several consequences for estimation, including (1) difficulty in selecting a definitive PLOD threshold score for discriminating between HSPs and third‐order kin and (2) requiring more restrictive assumptions, such as a stationary population and fixed reproductive schedules. However, subsistence harvest sampling is continuing, and sample sizes will increase. An additional 700 seals is expected to result in a doubling of the number of pairwise comparisons and observed kin pairs. Rerunning these models with additional data will improve reliability of PLOD threshold calculations, increase precision of the abundance estimate, improve insights into sex‐related differences in adult survival, refine the survival estimates, and establish trend estimates providing a robust approach for monitoring the status of a subsistence harvested marine mammal long‐term.

Incorporating age uncertainty into the model will result in more realistic estimates of precision for abundance and demographic parameters. Our CKMR models assume measured ages are exact. Although we recognize there is some uncertainty associated with aging, most of our ages were assigned the top‐quality code and we do not expect significant bias to result (though see Swenson et al. 2024). That said, including uncertainty in ages is possible in CKMR estimation provided there are external data on aging error (Bravington, Skaug, and Anderson 2016; Bravington, Grewe, and Davies 2016). External data could be acquired through blinded tooth aging experiments (e.g., Conn and Diefenbach 2007) or aging teeth from known‐age individuals. It is our intention to pursue such data and include aging uncertainty in a future model as sample sizes increase. Our model also assumes that there is no missing covariate (e.g., spatial location) that affects sampling probabilities of related individuals, though in practice CKMR is robust to limited violations of complete mixing and spatially balanced sampling (Conn et al. 2020). Practically speaking, we are assuming that we are not sampling a reproductively isolated portion of the population with elevated kinship frequencies relative to the whole population. The spatial distribution of our kin pairs supports this assumption.

Our CKMR models explicitly accounted for the possibility that GGPs are confounded with HSPs. Similar to the approach taken by Bradford et al. (2018), we modeled such observations as a mixture of the two kin pair types while accounting for inheritance of mtDNA as well as the sex of the unknown parent. While it is possible to eliminate GGPs by limiting kinship comparisons to those that have birth gaps less than 2*a* (*a* being the age of first birth), it reduces sample size and therefore precision relative to our modeling approach. The GGP‐HSP CKMR model should prove useful for researchers wishing to apply CKMR to other long‐lived species.

The fact that we did not find any FSPs greatly reduces the possibility of HSPs being contaminated by FTPs, which have the same expected proportion of co‐inherited DNA as HSP‐GGPs. The presence of mate fidelity can complicate CKMR modeling. If mate fidelity is rare, one might just ignore the few FTPs that may be present and get approximately unbiased inference, although the number of HSPs would be slightly overestimated and abundance underestimated. If mate fidelity is more common, then new and more complex models may be required, or kin may need to be restricted to POPs. This will need to be evaluated on a species‐by‐species basis, with larger sample sizes generally required when developing more complex models or relying solely on POPs.

The primary methods currently available for obtaining robust abundance estimates of wide‐ranging marine mammals in remote environments are aerial and ship‐based transect surveys, which are expensive. The cost of aerial surveys to cover the entire Beringia DPS are on the order of $1,000,000 USD (although aerial surveys provide information on three additional seal species). The genetic and analytical costs for this CKMR project to date were ~$100,000 USD not including personnel and sample collection costs. We estimate future genetic analytical costs to be ~$15,000 with each additional set of ~700 samples analyzed every 2–3 years. Depending on the availability of samples CKMR could have a substantial economic advantage over the cost of aerial surveys.

In this first successful application of CKMR to marine mammals, we were able to produce a reasonable abundance estimate that is comparable to that produced by aerial surveys, detect a possible sex‐related difference in adult survival, determine that fewer adult males participate in breeding than expected, and identify low mate fidelity even with our limited number of kin pairs. Additional samples are being collected and analyzed that will improve our results and provide trend in abundance going forward. Although tooth aging is the best method of aging for all age classes, there is variability and incorporating that uncertainty into the model will result in more realistic estimates of precision of abundance and survival. In Alaska, but also elsewhere in the world, marine mammals such as ice seals, walruses, beluga whales, and polar bears are harvested for subsistence purposes, and obtaining information to manage these resources has been difficult and expensive. CKMR, particularly when applied to harvest samples, is a cost‐effective means of providing reliable estimates of abundance and other demographic parameters, as well as life history information needed to properly balance conservation and subsistence needs.

## Conflicts of Interest

The authors declare no conflicts of interest.

## Supporting information


Appendix S1.

Appendix S2.

Appendix S3.

Appendix S4.

Appendix S5.

## Data Availability

A portion of the genetics data for this study is currently available at GenBank (GenBank PP178967.1 to PP179021.1). Additional genetics data, the model code, and data input files are permanently archived and publicly available in the Sequence Read Archive maintained by the National Center for Biotechnology Information and the DRYAD Digital Repository (https://doi.org/10.5061/dryad.z08kprrpw).
