# Supplementary material for: Estimating Demographic Parameters for Bearded Seals, Erignathus barbatus, in Alaska Using Close‐Kin Mark‐Recapture Methods
Source: Evol Appl. 2024 Nov 8;17(11):e70035. doi: 10.1111/eva.70035 (PMC11549065; doi:10.1111/eva.70035)
Supplement: Supplementary file 2 — Appendix S2. [file EVA-17-e70035-s001.docx]

**Supplement_2: CKMR DNA protocol**

Lab Supplies

- 10% bleach solution
- 95% ETOH
- DNA free 96 well plates
- DNA free 10 strip caps
- Forceps
- Sterile scalpel blades and handles
- Weight boats and scale
- Micropipette (70-100ml)
- Sterile pipet tips
- Chem wipes

**Organizing samples and datasheets:**

- To minimize errors, we create the sample order and datasheet before sampling. Each plate should have only one tissue type. We ordered by tissue, year, location, then ID. Once organized, we put the tissue samples into totes by plate and then bags in the tote by column.

**Subsampling**:

1. The utensils should be cleaned between samples with a 10% bleach solution, followed by ultra-pure clean water, and then 95% ETOH.
2. Using forceps as a prybar, cut 5-10mg (0.005-0.010g)* of partially frozen tissue without taking the tissue out of the plastic bag to minimize blood.
3. Place the tissue in a clean weight boat and weigh on scale (record weight in the spreadsheet).
4. Pick sample up from weight boat and deposit in correct well on 96 well plate.
5. Mark ID and well location on datasheet.
6. Once a column on the plate is full add ~100 ul of 95% ETOH (using a micropipette) to each well. Be sure the ETOH covers the whole sample but does not overfill the well.
7. Cap the row with strip caps (note direction of strip caps). Be sure caps are on tight and that liquid does not overflow from wells.
8. Sterilize utensils before starting new sample. Scalpel blades, weight boats, paper blotters, and pipet tips must be replaced for each sample.

**Filling Plates:**


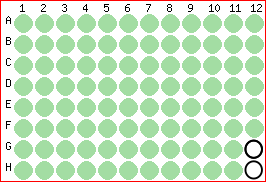


Leave blank

Start at A1 and fill down the column.

1. Fill the plate from left to right starting at the top in well A1. Fill down column 1 until all wells are filled. Add alcohol and then cap the column before moving onto the next column. The unfilled columns to the right of the active column should be covered with tinfoil to avoid contamination. If a plate is not completed in a work day, place strip caps over the empty wells.
2. Once filled plates are stored in the refridgerator until they are shipped. Mark the plate number and batch on the sides of the plate in sharpie.

* ***For a muscle sample of 5-10mg, the sample weight (with blood) should be ~6.5-10.5mg. For liver, the sample weight should be ~7-11mg. The liver samples leave more blood behind in the weight boats.***
