# Supplementary material for: Estimating Demographic Parameters for Bearded Seals, Erignathus barbatus, in Alaska Using Close‐Kin Mark‐Recapture Methods
Source: Evol Appl. 2024 Nov 8;17(11):e70035. doi: 10.1111/eva.70035 (PMC11549065; doi:10.1111/eva.70035)
Supplement: Supplementary file 3 — Appendix S3. [file EVA-17-e70035-s003.docx]

**Supplement_3: Kinship Determinations - Technical details**

*1. Hardy Weinburg equilibrium (HWE) quality control criterion.*

Apart from culling loci with low read counts, removing loci significantly out of HWE resulted in the largest reduction. Prior to HWE filtering we had 2,634 loci some of which had poor fit statistics (e.g., the pink loci in Fig. S3a). Experience has shown that rejecting loci based on “absolute” p-value (e.g., significant failure to fit HWE at p =1e-04) is unnecessarily conservative (and of course highly dependent on sample size, which is not sensible); a locus can be "significantly" out of HWE but still perfectly effective for kin-finding. However, a HWE p-value is still a useful relative criterion for distinguishing better loci from worse loci. Here, choosing a threshold of p > 1e-05 seems to effectively separate a group of systematically-mis-fitting loci (i.e., pink from non-pink in Fig. S3a).


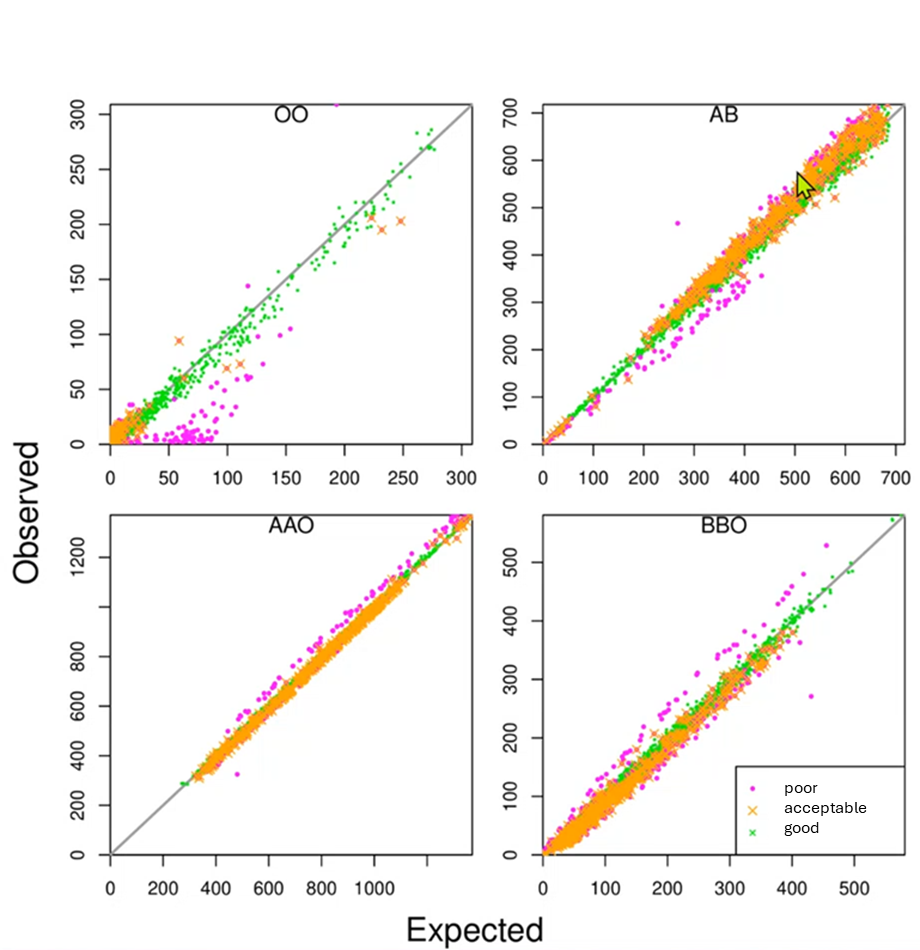


Figure S3a: HWE observed vs. expected plots. Each point is a locus and that same locus appears in each of the 4 panels. The y-axis is the number of samples for which each locus was observed as a double null 00, the homozygotes (AA or AO and BB or BO) or the heterozygote (AB). The x-axis is the number of samples for each of the four alleles expected under HWE. The line represents perfect fit to HWE. The loci are color coded for goodness of fit using a chi-square fit statistic. Here pink symbols (poor loci) have p-values < 10e-05. Each locus has a consistent color in each of the 4 panels. For more details regarding HWE filtering see the vignette to the R package “Kinference” (Bravington et al. 2021).

HWE filtering resulted in the removal of 612 loci and greatly improved the overall fit (Fig. S3b). The ultimate test of the whole QC process is whether the kin finding results agree with H-W theoretical distributions, which they do (See Section 2. Kinship Determinations Figs. S3c, S3d, and S3e).


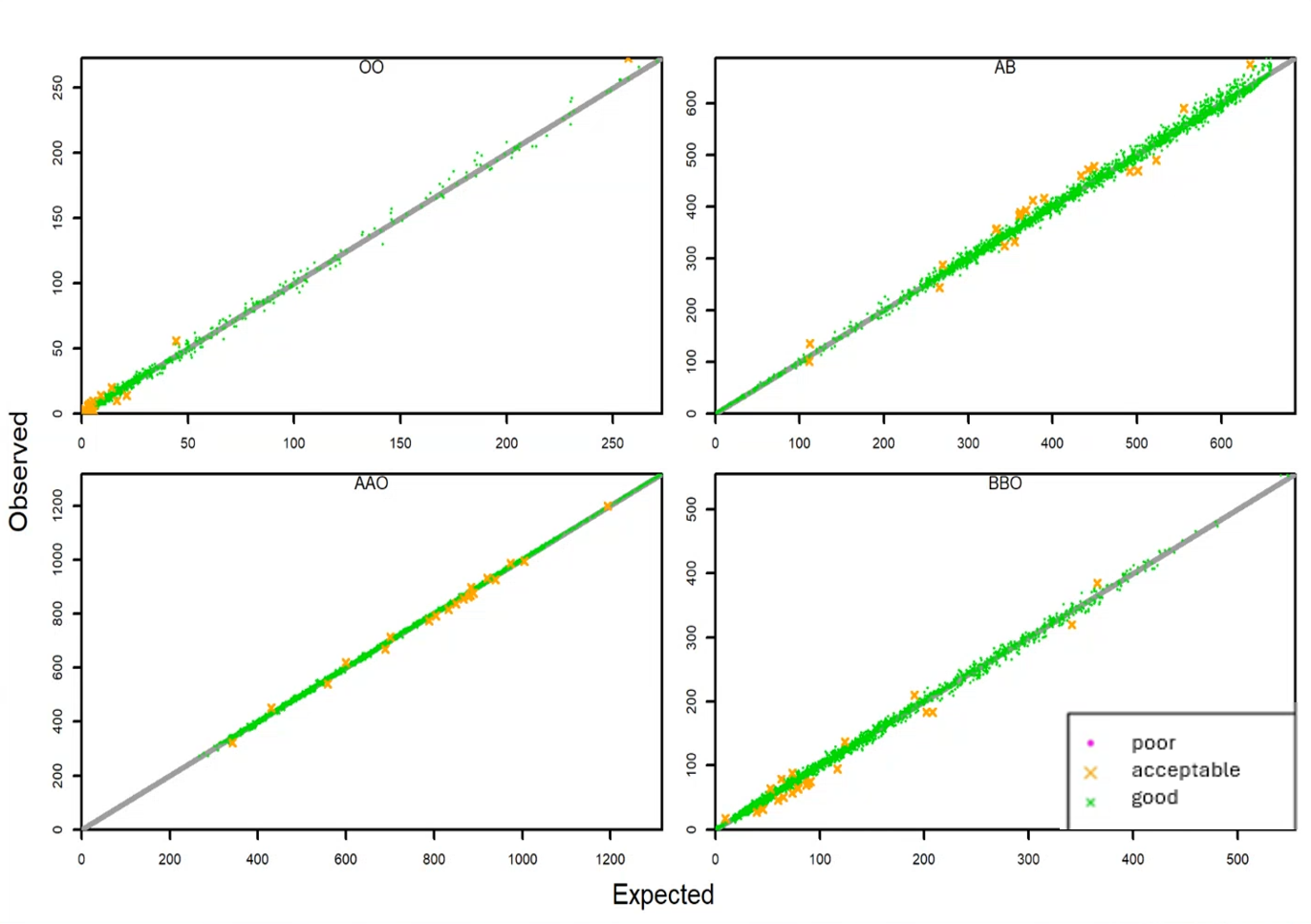


Figure S3b: HWE observed vs. expected plots. Each point is a locus and that same locus appears in each of the 4 panels. The y-axis is the number of samples for which each locus was observed as a double null 00, the homozygotes (AA or AO and BB or BO) or the heterozygote (AB). The x-axis is the number of samples for each of the four alleles expected under HWE. The line represents perfect fit to HWE. The loci are color coded for goodness of fit using a chi-square fit statistic. Each locus has a consistent color in each of the 4 panels.

2. *Kinship Determinations*

Kinship Inference followed the likelihood-based approach developed by Bravington et al. (2016) for calculating likelihood-ratio kin identification statistics. We calculated two types of Pseudo^^[[1]](#footnote-1)^^ log-odds ratio (PLOD) scores using functions in the Kinference R package (Bravington et al. 2021). To distinguish first-order kin including POPs and full sibling pairs (FSPs) from second-order kin such as HSPs and GGPs, we calculated HSP/UP PLOD scores for which unrelated pairs (UPs) was the null kin-type and HSPs (or any other second-order kin type) was the alternative. The distribution of HSP/UP PLOD scores depicts a large peak appropriately centered around the Hardy-Weinberg (H-W) theoretical mean for UPs (-78.5) with a variance consistent with that predicted (160.7), a smaller peak concentrated around the H-W theoretical mean for HSPs (+77.2), and first-order kin at substantially larger positive scores near +230 (Fig. S3c). Agreement between expectations predicted by statistical genetics theory and the observed distributions indicated that the genotyping and kin-finding worked properly (e.g., there were no cryptic populations and no serious genotyping errors) and provided confidence that the approach to estimate the number of second-order kin (described below) was reliable.


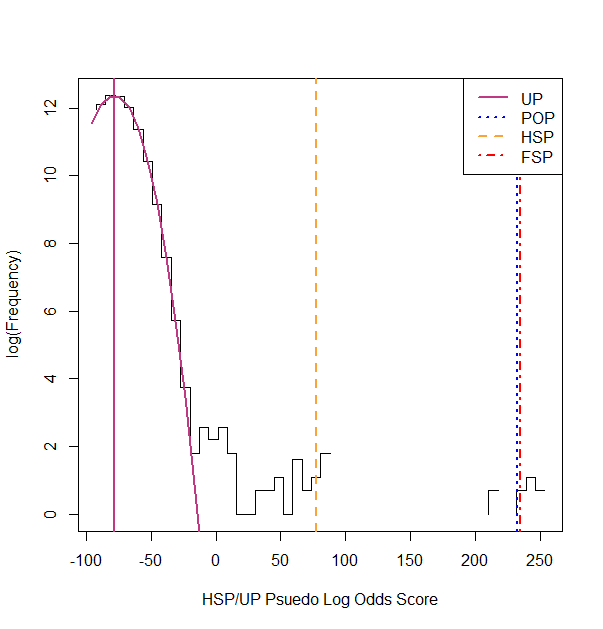


Figure S3c: Observed log-frequencies of HSP/UP PLOD scores for all pairwise comparisons. The log number of pairs for a binned PLOD value is displayed by the black histogram bars. The large peak at the left indicates the distribution of PLOD scores for unrelated pairs (UP), and the solid purple curve and vertical line at -78.5 depict the Hardy-Weinberg (H-W) theoretical distribution of PLOD scores and mean for unrelated pairs, respectively. At PLOD score ~ 77, there is a peak in the observed data around the H-W theoretical mean for HSPs (orange dashed vertical line). At PLOD scores near 230, two nearly coincident vertical lines (blue dotted and red dot-dashed) show the H-W theoretical means for parent-offspring pairs (POP) and full-sibling pairs (FSP). The close alignment of the observed data with the theoretical means for all these kin types and the variance of the UP distribution (160.7) is evidence that our set of genotypes is adequate for kin detection.

HSP/UP PLOD scores identified 11 first-order kin for which we calculated FSP/POP PLOD scores (Trenkel et al. 2022) to clearly distinguish POPs (large negative scores) from FSPs (large positive scores). All 11 are distributed around the H-W theoretical mean for POPs (-64.4) and far from the H-W theoretical mean for FSPs (+89.5) (Fig. S3d). Importantly, a lack of FSPs in our bearded seal sample simplified our approach to kin-finding. First, having no FSPs precludes confounding HSPs with FTPs, which have the same expected proportion of coinherited DNA as HSP-GGPs. Second, no FSPs precludes confounding HSPs with third-order kin whose presence requires the existence of FSPs (i.e., first (full) cousins and double first cousins). Thus, half thiatic pairs (HTPs, e.g., half aunt-niece) are left as the primary third-order kin that can be confounded with HSPs in our sample.


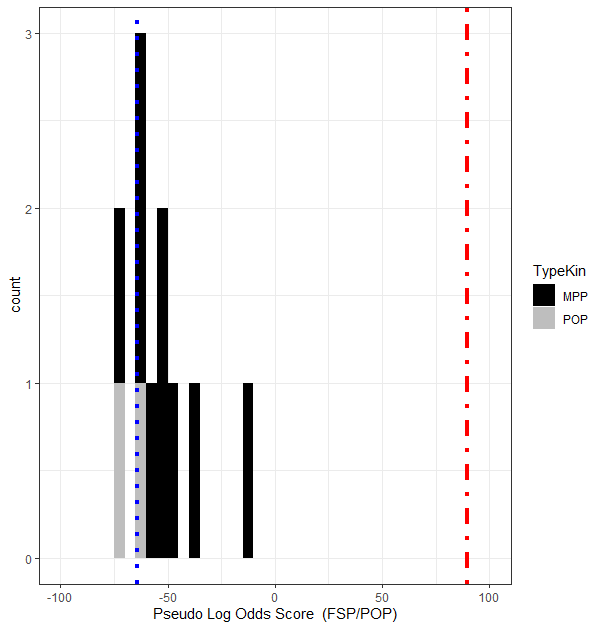


Figure S3d. Distinguishing POPs from FSPs. The distribution of FSP/POP PLOD scores is shown for the nine dependent mother/pup pairs (MMPs, black bars) and the two potential independent POPs (gray bars). The Hardy-Weinburg theoretical mean for POPs is -64.4 (blue dotted line) and that for FSPs is +89.5, far to the right of the MMPs with the largest PLOD scores (red dot dashed line). One of the two potential independent POPs has a PLOD score of -62.8, which is close to the POP mean of -64.4 and the second has a PLOD score of -72.3, which is the lowest PLOD score and farthest away from the distant FSP mean.

Distinguishing between second- and third-order kin such as HTPs cannot be done with certainty because scores for third-order kin lie between UPs and second-order kin partially overlapping with the latter (Fig. S3e). To estimate the number of HSP-GGPs we imposed a lower threshold for HSP/UP PLOD scores with the goal of eliminating false positive HSPs (i.e., counting third-order kin as an HSP-GGP) while simultaneously providing a way to estimate and compensate for false negatives (i.e., HSP-GGPs with HSP/UP PLOD scores less than the selected threshold). Provided that the threshold was set high enough to exclude nearly all third-order kin, CKMR inference will be robust to its precise location (Supplement_4). We accounted for the false negative HSP-GGPs by adjusting CKMR kinship formulae to include a false-negative loss probability. To compute that probability, which we treated as a fixed variable rather than an estimated parameter, we needed to estimate the variance ($\sigma^{2}$) of the HSP/UP PLOD scores for HSP-GGPs. Because the left-hand side of the PLOD score distribution was potentially contaminated by third-order kin, we used the right-hand side of the distribution to estimate $\sigma^{2}$ by fitting a half-normal distribution to these scores using maximum likelihood. We imposed three alternative threshold PLOD scores (30, 40, and 50) and, for each, calculated the probability of detecting and including an HSP-GGP in our modeling procedure as follows:

$d=\int_{x=Threshold}^{\infty} f\left( x;\mu,\sigma^{2} \right)dx$ (1)

where *f* (*x; µ, σ^2^*) is a Gaussian probability density function with mean *µ* (set to the theoretical H-W mean) and variance *σ^2^*. This value $d$ was ultimately incorporated into HSP kinship probabilities (see, e.g., Supplement_4).


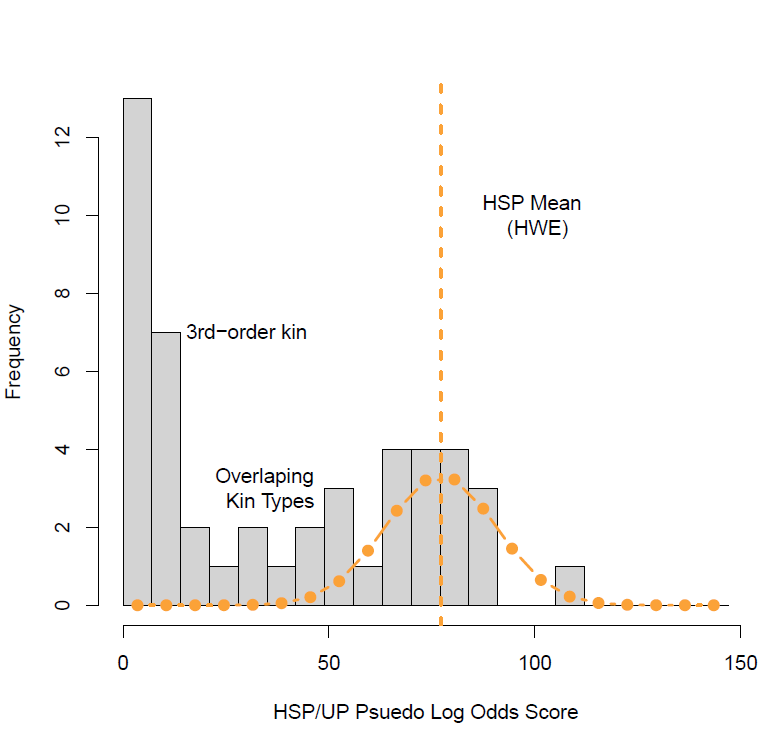


Figure S3e: Distinguishing HSPs from third-order kin. The distribution of HSP/UP PLOD scores is shown for kin pairs with HSP/UP PLOD scores between 0 and 150. The HSP distribution was estimated by first fitting a half normal distribution to the HSP values above the mean and using the result to project below the mean (details provided in text). The H-W theoretical mean for HSPs has a PLOD score ~77.2 which aligns reasonably well with the observed data peak.

In Fig. S3e, the orange dashed vertical line at HSP/UP PLOD score ~ 77.2 is the H-W theoretical mean for HSPs and GGPs, which aligned well with the observed peak of potential HSP-GGPs PLOD scores. The peak closer to zero was consistent with third-order kin, whose expected HSP/UP PLOD score is close to 0. There is a suggestion of a “valley” at scores of 21 to 49, but no clear separation of kin types.

LITERATURE CITED

Bravington, M. V., Miller, D. L. & Baylis, S. M. (2021). Kinference: pair wise kin-finding for close-kin mark-recapture. *R package version 0.0.80.*

Bravington, M., Thomson, R., & Davies, C. (2017). Genotyping issues for CKMR on Atlantic bluefin tuna. Atlantic-wide research programme on Bluefin Tuna (ICCAT GBYP PHASE 5) GBYP xxx/2016. International Commission for the Conservation of Atlantic Tuna.

Bravington, M. V., Skaug, H. J., & Anderson, E. C. (2016). Close-kin mark-recapture. *Statistical Science*, *31*(2), 259–274. <https://doi.org/10.1214/16-STS552>

Trenkel, V. M., Charrier, G., Lorance, P., & Bravington, M. V. (2022). Close-kin mark-recapture abundance estimation: Practical insights and lessons learned. *ICES Journal of Marine Science*, *79*(2), 413–422. [https://doi.org/10.1093/icesj ms/fsac002](https://doi.org/10.1093/icesj%20ms/fsac002)

1. The "Pseudo" in PLOD refers to how the statistic treats coinheritance as an independent event at each locus, which (except for unrelated pairs and POPs) it is not, due to physical linkage (Bravington et al. 2017). [↑](#footnote-ref-1)
