# Supplementary material for: Estimating Demographic Parameters for Bearded Seals, Erignathus barbatus, in Alaska Using Close‐Kin Mark‐Recapture Methods
Source: Evol Appl. 2024 Nov 8;17(11):e70035. doi: 10.1111/eva.70035 (PMC11549065; doi:10.1111/eva.70035)
Supplement: Supplementary file 4 — Appendix S4. [file EVA-17-e70035-s002.docx]

**Supplement_4: Formulation of the likelihoods for the HSP-GGP-POP CKMR model**

In this supplement, we describe the inferential framework for our HSP-GGP-POP CKMR model. Overall, inference was based on a maximum marginal pseudo-likelihood (MPL), with an observation model based on a product Bernoulli likelihood reflecting many pairwise kinship comparisons (Bravington et al. 2016). Specifically, we will base inference on the joint pseudo-likelihood:

*L* = *L_pop_L_hsp/ggp_f* (*η*)Λ_λ_,

where *L_pop_* is a product Bernoulli likelihood for POPs, *L_hsp/ggp_* is a product Bernoulli likelihood for HSPs and GGPs, *f* (*η*) are penalties on RAW survival parameters if they deviate from their prior mean (essentially, taking the place of a prior distribution), and Λ_λ_ is a penalty on λ values deviating from λ_0_, which is set by the analyst.

The pseudo-likelihood for POP comparisons (*L_pop_*) is a product Bernoulli distribution, specified as

$$L_{pop}=\prod_{i} \prod_{j} p_{ij1}^{y_{ij1}}\left( 1-p_{ij1} \right)^{1-y_{ij1}}I_{1}\left( i,j \right)$$

Here, *i* and *j* index two individuals, *y_ij1_* is a binary random variable that equals 1 if *i* and *j* are POPs and equals zero otherwise. *I*_1_(*i, j*) is an indicator function used to prevent double counting, and to omit certain comparisons that are likely to violate independence assumptions for the Bernoulli model. For instance, we set *I*_1_(*i, j*) = 0 whenever the year of *i*’s birth (*b_i_*) is greater than or equal to the year of *j*’s birth (*b_j_*); we also set *I*_1_(*i, j*) = 0 whenever (1) *i* is female and (2) *i* and *j* are both harvested in the year of *j*’s birth (i.e., *d_i_* = *d_j_* = *b_j_*). The latter restriction is to prevent dependency in harvests of mothers and pups, which can negatively bias CKMR abundance estimates. We term this a pseudo-likelihood because it technically assumes independence between kin comparisons, even though there is necessarily some dependence among animals (e.g., a seal cannot have two mothers). Nevertheless, the Bernoulli approximation to the true likelihood has been shown to perform well with large populations (including variance calculations based on second derivatives; Bravington et al. 2016).

For HSPs and GGPs, the pseudo-likelihood *L_hsp/ggp_* is again a product Bernoulli,

$$L_{hsp/ggp}=\prod_{i} \prod_{j} p_{ij2}^{y_{ij2}}\left( 1-p_{ij2} \right)^{1-y_{ij2}}I_{2}\left( i,j \right)$$

where the “2” subscript simply denotes that data, success probabilities, and constraints are particular to HSPs and GGPs. In particular, we have $p_{ij2}=dp_{ij}\left( HSP \right)+dp_{ij}(GGP)$, where $p_{ij}\left( HSP \right)$ and $p_{ij}(GGP)$ are the probabilities of *i* and *j* being HSPs or GGPs, respectively, and *d* is the complement of the false negative probability (which is input as a fixed variable, not an estimated parameter; see main article text for details of its calculation). Here, we again set *I*_2_(*i, j*) = 0 whenever *b_i_* > *b_j_* to prevent double counting. Note that additivity of HSP and GGP probabilities is justified since the associated events are mutually exclusive.

We now describe the probability that two individuals, selected at random, are either a half-sibling pair (HSP), a grandparent-grandchild pair (GGP), or a parent-offspring pair (POP) conditional on relevant covariates. For purposes of these calculations, covariates consist of ages (and applied birthdates), sex, date of harvest, and mtDNA haplotype.

***Parent-offspring pair probabilities:***

Calculations of *p_ij_*_1_ differ based on whether the potential parent is male or female. In addition to reproductive output being based on maturity-at-age for males and fecundity-at-age for females, there is also the issue of timing of reproduction and pupping. Specifically, females need to survive the whole annual reproductive cycle to the spring when their pup is born, while males only need to survive to breed in May-June to sire a pup. In general, the *p_ij_* are based on the concept of expected relative reproductive output (ERRO; Bravington et al. 2016), which is the expected reproductive output of a potential parent in the year of the offspring’s birth relative to the total reproductive output of the population. Total reproductive output, $T_{s}\left( y \right)$, varies by sex (*s*) and year (*y*), such that

$T_{0}\left( b_{j} \right)= \sum_{a} N_{b_{j}}^{F}f_{a}$for females (*s* = 0), and

$T_{1}\left( b_{j} \right)= \sum_{a} N_{b_{j}-1}^{M}m_{a}$for males (*s* = 1), (Eqn. S1)

where *f_a_* is fecundity-at-age and *m_a_* is male maturity-at-age, respectively.

Following this definition, we have:

$$p_{ij1}=\left\{ \begin{aligned} f_{b_{j}-b_{i}}T_{0}^{-1}\left( b_{j} \right) \mathrm{if} d_{i}\geq b_{i}&& &&and& {(b}_{j}-b_{i})<40 \mathrm{and} s_{i}=0 \\ m_{b_{j}-b_{i}-1}T_{1}^{-1}\left( b_{j} \right) \mathrm{if} d_{i}\geq b_{i}&& &&and {(b}_{j}-b_{i})<40 \mathrm{and} s_{i}=1 \\ 0 otherwise. \end{aligned} \right.$$

Note that we include the (*b_j_* − *b_i_*) < 40 restriction because our population model is restricted to ages 0-39 and parameters are undefined past this range. Our priors on survival suggest that few bearded seals would survive long enough for this to matter.

***Half sibling pair probabilities:***

For HSPs, we never directly observe the common parent, although we assume that we know what sex it is because maternally related HSPs (MHSPs) share mtDNA. Technically, it is possible that paternally related HSPs (PHSPs) could have the same mtDNA by random chance, but the probability is low (e.g., ≈ 1%) because mitochondrial haplotype diversity is quite high for bearded seals (Quakenbush and Sheffield 2007, Lang et al. 2016, and Lang et al. 2017). The probabilities $p_{ij}\left( HSP \right)$ once again reflect relative reproductive output, but we must sum over possible parent ages because we never observe the parent directly, accounting for whether the parent shares ($h_{ij}=1)$ or does not share ($h_{ij}=0)$ mtDNA. Also, the prospective parent must survive from *b_i_* → *b_j_* for females, and from *b_i_* → *b_j_*−1 for males. Once again, we need to have age restrictions to prevent the potential parent from achieving an age ≥ 40 where parameters are undefined. Letting *δ_ij_* = *b_j_* − *b_i_* and by doing some algebra, we have

$$p_{ij}(HSP)=\left\{ \begin{aligned} \frac{\sum_{a=0}^{40-\delta_{ij}} N_{b_{i}-1, a}^{M}m_{a}m_{a+\delta_{ij}}\prod_{c=a}^{a+\delta_{ij}-1} \varphi_{c}}{\left\{ \sum_{c} N_{b_{i}-1, c}^{M}m_{c} \right\}\left\{ \sum_{c} N_{b_{j}-1, c}^{M}m_{c} \right\}} \text{if} h_{ij}=0 \\ I_{ij}\frac{\sum_{a=0}^{40-\delta_{ij}} N_{b_{i}, a}^{F}f_{a}f_{a+\delta_{ij}}\prod_{c=a}^{a+\delta_{ij}-1} \varphi_{c}}{\left\{ \sum_{c} N_{b_{i}, c}^{F}f_{c} \right\}\left\{ \sum_{c} N_{b_{j}, c}^{F}f_{c} \right\}} \text{if} h_{ij}=1. \end{aligned} \right.$$

Substitutions for ERRO are as follows:

For female parents ($h_{ij}$=1)

$$T_{0}\left( b_{i,c} \right)= \sum_{c} N_{b_{i,c}}^{F}f_{c} \text{and }T_{0}\left( b_{j,c} \right)= \sum_{c} N_{b_{j,c}}^{F}f_{c}$$

For male parents ($h_{ij}$=0)

$$T_{1}\left( b_{i,c} \right)= \sum_{c} N_{b_{i-1`,c}}^{M}m_{c}\text{and }T_{1}\left( b_{j,c} \right)= \sum_{c} N_{b_{j-1,c}}^{M}m_{c}$$

Resulting in

$p_{ij}(HSP)=\left\{ \begin{aligned} \frac{\sum_{a=0}^{40-\delta_{ij}} N_{b_{i}-1, a}^{M}m_{a}m_{a+\delta_{ij}}\prod_{c=a}^{a+\delta_{ij}-1} \varphi_{c}}{T_{1}\left( b_{i,c} \right)T_{1}\left( b_{j,c} \right)} \text{if} h_{ij}=0 \\ I_{ij}\frac{\sum_{a=0}^{40-\delta_{ij}} N_{b_{i}, a}^{F}f_{a}f_{a+\delta_{ij}}\prod_{c=a}^{a+\delta_{ij}-1} \varphi_{c}}{T_{0}\left( b_{i,c} \right)T_{0}\left( b_{j,c} \right)} \text{if} h_{ij}=1. \end{aligned} \right.$ (Eqn. S2)

The indicator *I_ij_* = 1 if δ_ij_ > 0 and is used to disallow MHSPs from occurring if birth years of the prospective HSP kin pairs are the same (because females only have one pup per year). For males, whenever *δ_ij_* = 0, we set $\prod_{c=a}^{a+\delta_{ij}-1} \varphi_{c}=1.0$. This formulation is similar to that reported by Hillary et al. (2018) for analysis of white shark HSPs, although that application was rendered more complicated by larger litter sizes and multiple within-litter paternity.

***Grandparent-grandchild pair probabilities:***

We shall assume that mtDNA haplotype diversity is high enough that a grandparent and grandchild will only share mtDNA if (1) the potential grandparent is female, and (2) the unobserved parent of the grandchild (the grandparent’s direct offspring) is female. This dynamic can be visualized in Fig. S4a.


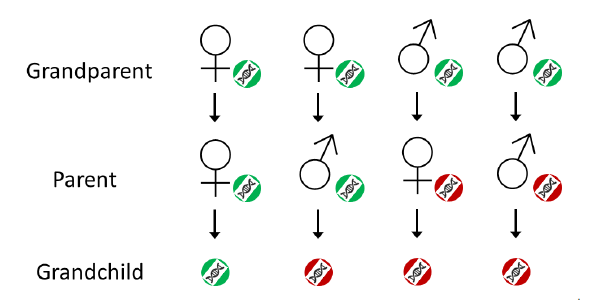


**Figure S4a**

Here, the green DNA bits show inheritance of mtDNA, with green mtDNA being identical to that possessed by the grandparent. Importantly, if the potential grandparent is male, the chance that the GGP shares mtDNA is negligible (this would not be the case in populations with low mtDNA haplotype diversity).

There are four different possibilities, associated with (1) the sex of the parent, and (2) whether or not the two individuals compared share mitochondrial DNA. We shall describe each of these cases separately, letting *s_i_* denote sex of the older seal (with *s_i_* = 1 if *i* is male), and again letting *h_ij_* be a binary random variable that takes on the value 1 if individuals *i* and *j* share the same mtDNA haplotype. As above, substitutions emphasizing ERRO could be made in the equations for each of the cases below.

**Case 1:** *s_i_* = 0, *h_ij_* = 1

We’ll denote the probability of a GGP sharing mtDNA when the potential grandparent is female as $Pr(GGP,h_{ij}=1|s_{i}=0,d_{i},b_{i}, b_{j})$. Note that this expression depends on the time of death of the potential grandparent, $d_{i}$ (for instance, if it dies before it was old enough to have potentially reproduced, it is clearly not a grandparent), and birth years of the potential grandparent and grandchild ($b_{i}$ and $b_{j}$, respectively). It is also dependent on female fecundity-at-age ($f_{a}$), year- and age-specific adult abundance ($N_{t,a}$), and age-specific survival probability ($\phi_{a}$). Note also that we are assuming (by virtue of high mtDNA haplotype diversity) that a grandparent and grandchild can only share mtDNA if the unobserved parent is female. Accordingly,

$$\Pr\left( GGP,h_{ij}=1 | s_{i}=0,d_{i},b_{i}, b_{j} \right)=\sum_{t=b_{i}}^{\text{min}(d_{i},b_{j})} \frac{f_{t-b_{i}}N_{t,0}}{\sum_{a} f_{a}N_{t,a}}\frac{\left\{ \prod_{k=t}^{b_{i}-1} \varphi_{k-t} \right\}2f_{b_{j}-t}}{\sum_{a} f_{a}N_{b_{j},a}}=\sum_{t=b_{i}}^{\text{min}(d_{i},b_{j})} \frac{f_{t-b_{i}}N_{b_{j},b_{j}-1}}{\sum_{a} f_{a}N_{t,a}}\frac{2f_{b_{j}-t}}{\sum_{a} f_{a}N_{b_{j},a}}$$

Here, relative reproductive success is conditional on the unknown age of the parent, so we must sum over the possible years (*t*) of the mother’s birth. The ($N_{b_{j},b_{j}-t}$) in the numerator arises because the potential parent can be any of the females born in year *t* that survive to the year of *j*’s birth. For seals, many of the *f_a_* values are zero for low ages, so practically speaking we must have a sufficient birth gap (and late enough time of death for the potential grandparent) to enable *Pr*(*GGP* ) *>* 0. As in previous calculations, this formulation requires a number of things to hold like equal male:female sex ratios, equal survival among sexes, etc.

**Case 2:** *s_i_* = 0*,* $h$*_ij_* = 0

The only way for a grandmother and grandchild not to share mtDNA is if the unobserved parent is male, so our answer will be similar but will involve male maturity-at-age indexed to the year before *j*’s birth:

$$\Pr\left( GGP,h_{ij}=0 | s_{i}=0,d_{i},b_{i}, b_{j} \right)=\sum_{t=b_{i}}^{\text{min}(d_{i},b_{j}-1)} \frac{f_{t-b_{i}}}{\sum_{a} f_{a}N_{t,a}}\frac{N_{b_{j}-1, b_{j}-t-1}2m_{b_{j}-t-1}}{\sum_{a} m_{a}N_{b_{j}-1,a}}$$

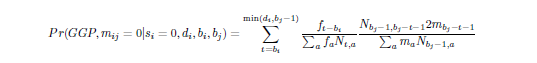


**Case 3:** *s_i_* = 1*,* $h$*_ij_* = 1

By assumption, $\Pr\left( GGP,h_{ij}=1 | s_{i}=1,d_{i},b_{i}, b_{j} \right)=0$ for reasons stated previously.

**Case 4:** *s_i_* = 1*,* $h$*_ij_* = 0

This case can happen whether the offspring of *i* is male or female, so we must account for both. Fortunately, it is very similar to what we have written already, though it involves male maturity in the year before the birth of the prospective parent:

$$\Pr\left( GGP,h_{ij}=0 | s_{i}=1,d_{i},b_{i}, b_{j} \right)=\sum_{t=b_{i}+1}^{\text{min}(d_{i},b_{j})} \frac{m_{t-b_{i}-1}}{\sum_{a} m_{a}N_{t-1,a}}\frac{N_{b_{j}, b_{j}-t}2f_{b_{j}-t}}{\sum_{a} f_{a}N_{b_{j},a}}=\sum_{t=b_{i}+1}^{\text{min}(d_{i},b_{j})} \frac{m_{t-b_{i}-1}}{\sum_{a} m_{a}N_{t-1,a}}\frac{N_{b_{j}-1, b_{j}-t-1}2m_{b_{j}-t-1}}{\sum_{a} m_{a}N_{b_{j},a}}$$

**Additional objective function components**

The last two terms in the MPL are *f* (*η*) and Λ_λ_. For *f* (*η*), we specified independent Gaussian prior distributions for RAW parameters, with a mean set to the values estimated from hierarchical meta-analysis (Trukhanova, Conn, and Boveng 2018), and with a standard deviation set to achieve a coefficient of variation (CV) of approximately 0.2 on the real scale. For Λ_λ_, a Gaussian penalty was set on the realized finite rate of population growth λ, such that λ ∼ Normal (λ_0_, 10^−8^). The small variance ensured that the realized finite rate of population increase would be close to λ_0_ (which was set to 1.0 in most models). Log link functions were used on all parameters (abundance, RAW parameters) to constrain real-valued estimates to be positive.

**Modifications for heterogeneity in male reproductive success**

To modify the previous formulae to account for possible heterogeneity in male breeding success, we incorporated an additional parameter, $\pi$, $0<\pi\leq1$, that represents the fraction of breeding age males that successfully reproduce. The modification is simply to replace $N_{t}^{M}$with ${\pi N}_{t}^{M}$ everywhere it exists. For instance, Eqn. S1 becomes:

$T_{1}\left( b_{j} \right)= \sum_{a} {\pi N}_{b_{j}-1}^{M}m_{a}$,

and the first case of Eqn. S2 becomes

$\frac{\sum_{a=0}^{40-\delta_{ij}} {\pi N}_{b_{i}-1, a}^{M}m_{a}m_{a+\delta_{ij}}\prod_{c=a}^{a+\delta_{ij}-1} \varphi_{c}}{T_{1}\left( b_{i,c} \right)T_{1}\left( b_{j,c} \right)}$, etc.

LITERATURE CITED

Bravington, M. V., Skaug, H. J., & Anderson, E. C. (2016). Close-kin mark-recapture. *Statistical Science*, *31*(2), 259–274. <https://doi.org/10.1214/16-STS552>

Hillary, R. M., Bravington, M. V., Patterson, T. A., Grewe, P., Bradford, R., Feutry, P., ... & Bruce, B. D. (2018). Genetic relatedness reveals total population size of white sharks in eastern Australia and New Zealand. *Scientific reports*, 8(1), 2661.

Lang, A. R., Quakenbush, L., Ziel, H., Robertson, K., Lauf, M., & Boveng, P. (2017). Detecting population structure in bearded and ringed seals: current understanding and future challenges. *Alaska Marine Science Symposium*. Anchorage, AK.

Lang, A., Quakenbush, L., & Boveng, P. (2016). Assessing the genetic structure of Arctic ice seals. Final Programmatic Report Narrative. Grant ID# 45916. 2015 Alaska Fish and Wildlife Fund Grant Awards. National Fish and Wildlife Foundation. Available from Marine Mammal Laboratory, NOAA Alaska Fisheries Science Center, Seattle, WA. 9 pp.

Quakenbush, L., & G. Sheffield. (2007). Ice seal bio-monitoring in the Bering-Chukchi Sea region. North Pacific Research Board (NPRB) Project 312 Final Report, 47 p. Alaska Department of Fish and Game, Fairbanks, AK.

Trukhanova, I. S., Conn, P. B., & Boveng, P. L. (2018). Taxonomy-Based Hierarchical Analysis of Natural Mortality: Polar and Subpolar Phocid Seals. *Ecology and Evolution*, 8(21), 10530–10541.
